# Structure of graphene and its disorders: a review

**DOI:** 10.1080/14686996.2018.1494493

**Published:** 2018-08-29

**Authors:** Gao Yang, Lihua Li, Wing Bun Lee, Man Cheung Ng

**Affiliations:** The State Key Laboratory of Ultraprecision Machining Technology, Department of Industrial and Systems Engineering, The Hong Kong Polytechnic University, Kowloon, Hong Kong

**Keywords:** Graphene, 2D materials, structure, disorder, defects modulation, review, 10 Engineering and Structural materials, 105 Low-Dimension (1D/2D) materials, 104 Carbon and related materials, 302 Crystallization / Heat treatment / Crystal growth

## Abstract

Monolayer graphene exhibits extraordinary properties owing to the unique, regular arrangement of atoms in it. However, graphene is usually modified for specific applications, which introduces disorder. This article presents details of graphene structure, including sp^2^ hybridization, critical parameters of the unit cell, formation of σ and π bonds, electronic band structure, edge orientations, and the number and stacking order of graphene layers. We also discuss topics related to the creation and configuration of disorders in graphene, such as corrugations, topological defects, vacancies, adatoms and sp^3^-defects. The effects of these disorders on the electrical, thermal, chemical and mechanical properties of graphene are analyzed subsequently. Finally, we review previous work on the modulation of structural defects in graphene for specific applications.

## Graphical Abstract

**Figure:**
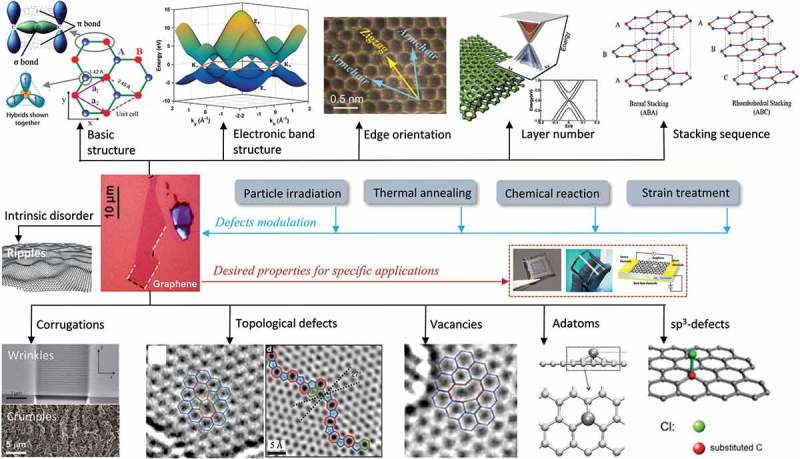

## 1. Introduction

The first study on graphene, or two-dimensional graphite, can be dated to as early as 1947 when Wallace used the ‘tight binding’ approximation to investigate the electronic energy bands in crystalline graphite [1]. Since it was shown that the semi-metallic phase is unstable in two dimensions [2,3], free standing monolayer graphene has long been regarded as an ‘academic’ material. Even so, many experimental efforts were made to obtain monolayer graphene. For instance, the monolayer graphene structures produced by hydrocarbon decomposition were observed on the Pt(111) surface under a scanning tunneling microscope (STM) in the early 1990s [4]. In 1997, Japanese scientists cleaved a kish graphite for the purpose of evaluating the thickness effect of graphite crystals on electrical properties; they successfully reduced the thickness of graphite films to 30 nm [5]. Inspired by this work, Novoselov and Geim presented a robust and reliable approach [6] for producing monolayer graphene by repeatedly peeling highly oriented pyrolytic graphite (HOPG) in 2004. The demonstration of mechanical exfoliation method, also called the scotch tape method, caused a great sensation and stimulated many scholars to investigate the structure and properties of graphene.

As a single atomic plane of carbon, graphene can be wrapped up into other graphitic materials such as fullerene, carbon nanotubes and thin graphene films [7]. Due to the internal exceptionally high crystal quality [8,9] and massless Dirac fermions [10], monolayer graphene exhibits anomalous half integer quantum Hall effect [11], remarkable optical properties [12,13], ultra-high intrinsic strength [14], superior thermal conductivity [15] and extremely high charge carrier mobility [6,16,17]. It is referred to as a zero-gap semiconductor, showing an exceptionally high concentration of charge carriers and ballistic transport because of the unique Dirac cone band structure near the Fermi level. Moreover, the propagation of massless electrons through the honeycomb lattice in a sub-micrometer distance without scattering makes it possible to investigate the quantum effects in graphene even at room temperature [18].

Graphene film consisting of few layers was employed to fabricate transistors, due to its strong ambipolar electric field effect [6]. However, the performance of the graphene transistor was limited because of the low on-off resistance ratio (less than ~ 30 at room temperature) that resulted from the thermally excited carriers. Fortunately, the properties of graphene can be tuned by preparing graphene sheets using different approaches or by incorporating graphene sheets in different materials, rendering a path to a broad new class of graphene-based composites.

**Figure 1.**
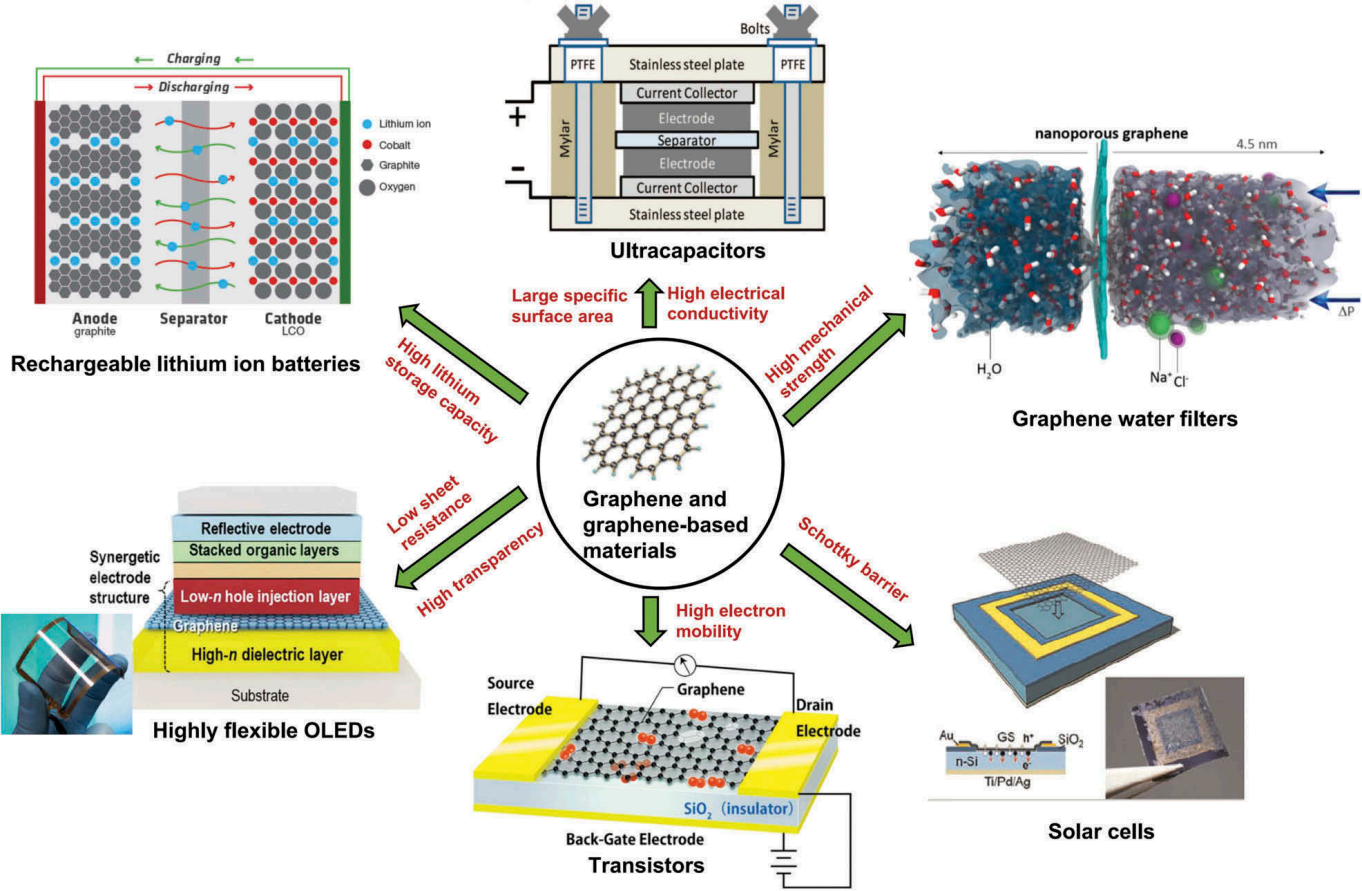
Applications of graphene and graphene-based materials in batteries, ultracapacitors [20], water filters [43], solar cells [44], transistors and OLEDs [26] (reused with permissions from [20] Copyright © 2008, American Chemical Society, [43] Copyright © 2012, American Chemical Society, and [44] Copyright © 2010, John Wiley and Sons.).

Graphene-based materials are expected to be promising building blocks in nanotechnology due to their varieties of applications, as seen in Figure 1. For example, by optimization of the structure (i.e. interlayer spacing, thickness and morphology of graphene nanosheets) and by incorporation of carbon nanotubes or C_60_ molecules, the lithium storage capacity of graphene nanosheets could be increased up to above 700 mAh/g, making them suitable for use in rechargeable lithium ion batteries [19]. The large surface area (2630 m^2^/g for a single graphene sheet) and high electrical conductivity (~ 200 S/m) gave a typical chemically modified graphene good performance in double layer capacitors [20]. A large-area graphene film with high electrical quality was grown on a copper substrate by chemical vapor deposition (CVD), and it was successfully used to fabricate dual-gated field effect transistors, with Al_2_O_3_ as the gate dielectric [21]. Quartz wafers, spin-coated by graphene oxide (GO) and annealed at a high-temperature (e.g. 1000 °C), exhibited a high-transparency, and showed great potential as heating, defrosting and antifogging devices [22]. The batch production of large-area, uniform graphene films on solid glass was realized by a catalyst-free atmospheric-pressure CVD approach [23]. Such graphene coated glass held good promise for thermochromic windows that benefit from the light interference effect of the changing layer thickness [24]. Due to the excellent bendability and high electrical conductivity, graphene can be adopted as an appropriate transparent electrode material [17,25]. The unique optical and electrical properties allows graphene to be applied to various optoelectronic devices, ranging from solar cells to touch screens [26]. Besides, graphene-based materials exhibit potential applications in catalysis [27], owing to their high specific surface area and accessibility to the surface; in gas sensors [28], owing to the sensitivity and selectivity of graphene towards various gas molecules; in waste energy harvesting [29], because of their unique shape and characteristics such as superconductivity, light weight, high-stiffness and axial strength; in protective coatings [30–35] owing to the high intrinsic mechanical strength and anti-corrosion ability; and in antibacterial packing [36]. More recent and comprehensive studies regarding applications of graphene can also be found in these reviews [37–42].

The aforementioned applications are realized by the modification of graphene on the basis of defects modulation, during which specified types of disorders are quantitatively created for altering the crystal structure of graphene and consequently obtaining the wanted properties. Therefore, having a good understanding of the graphene structure and its contained disorders is necessary for defects modification. Although several review articles have already introduced the morphology and structure of graphene [46,47], and discussed the lattice defects in graphene [48–50], this review provides background knowledge of intrinsic structure of graphene before the introduction of disorders so that the function of disorders in graphene lattice can be more easily understood. Moreover, this review has added some content on recent studies regarding graphene. In this review, the basics of the graphene structure, electronic band structure of graphene, edge orientations in graphene, number and stacking sequences of graphene layers are initially introduced. Subsequently, the disorders that are commonly seen in the graphene structure, including corrugations, topological defects, vacancies, adatoms and sp^3^-defects, are discussed separately. Besides, the formation energy and migration energy of different types of structural defects are briefly introduced, and the effects of defects on properties of graphene and the generation of disorders during graphene preparation procedures are analyzed. Finally, previous studies on defects modulation of graphene by various approaches (e.g. particle irradiation, thermal annealing, chemical reaction and strain treatment) are reviewed.

## 2. The structure of graphene

### 2.1. Basics of graphene structure

**Figure 2.**
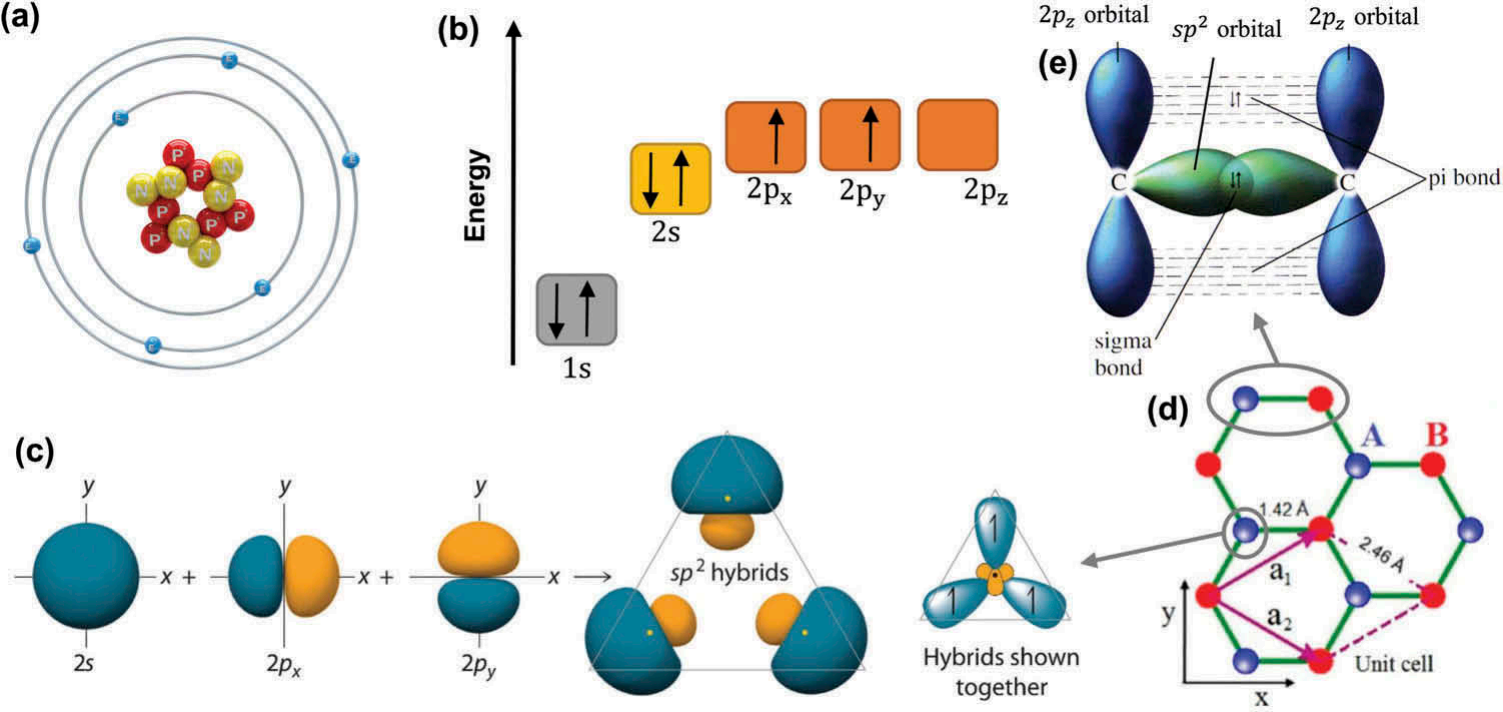
(a) Atomic structure of a carbon atom. (b) Energy levels of outer electrons in carbon atoms. (c) The formation of sp^2^ hybrids. (d) The crystal lattice of graphene, where A and B are carbon atoms belonging to different sub-lattices, a_1_ and a_2_ are unit-cell vectors. (e) Sigma bond and pi bond formed by sp^2^ hybridization.

Carbon is the sixth element in the periodic table, with a ground-state electronic configuration of $1s^{2}2s^{2}2{P_{x}}^{1}2{P_{y}}^{1}2{P_{z}}^{0}$, as shown in Figure 2(b). For convenience, the energy level of $2p_{z}$ is kept with no electron, though it is equivalent to the energy levels of $2p_{x}$ and $2p_{y}$. As seen in Figure 2(a), the nucleus of a carbon atom is surrounded by six electrons, four of which are valence electrons. These electrons in the valence shell of a carbon can form three types of hybridization, namely sp, sp^2^ and sp^3^. Figure 2(c) illustrates the formation of sp^2^ hybrids. When carbon atoms share sp^2^ electrons with their three neighboring carbon atoms, they form a layer of honeycomb network of planar structure, which is also called monolayer graphene. The unit cell of a graphene crystal, marked by a purple parallelogram in Figure 2(d), contains two carbon atoms, and the unit-cell vectors a_1_ and a_2_ have the same lattice constant of 2.46 Å. The resonance and delocalization of the electrons are responsible for the stability of the planar ring.

In a typical sp^2^ hybridization of two neighboring carbon atoms on the graphene layer (see Figure 2(e)), an out-of-plane π bond is made up by $2p_{z}$ orbitals which are perpendicular to the planar structure, while an in-plane σ bond is formed by the sp^2^ (2s, $2p_{x}$ and $2p_{y}$) hybridized orbitals. The resulting covalent σ bond has a short interatomic length of ~ 1.42 Å, making it even stronger than the sp^3^ hybridized carbon–carbon bonds in diamonds, thus giving the monolayer graphene remarkable mechanical properties (e.g. a Young’s modulus of 1 TPa and an intrinsic tensile strength of 130.5 GPa [14]). In monolayer graphene, the conduction band and valence band with zero band gap are formed due to the half-filled π band that permits free-moving electrons. Further, the π-bonds provide a weak van der Waals interaction between adjacent graphene layers in bilayer and multi-layer graphenes.

**Figure 3.**
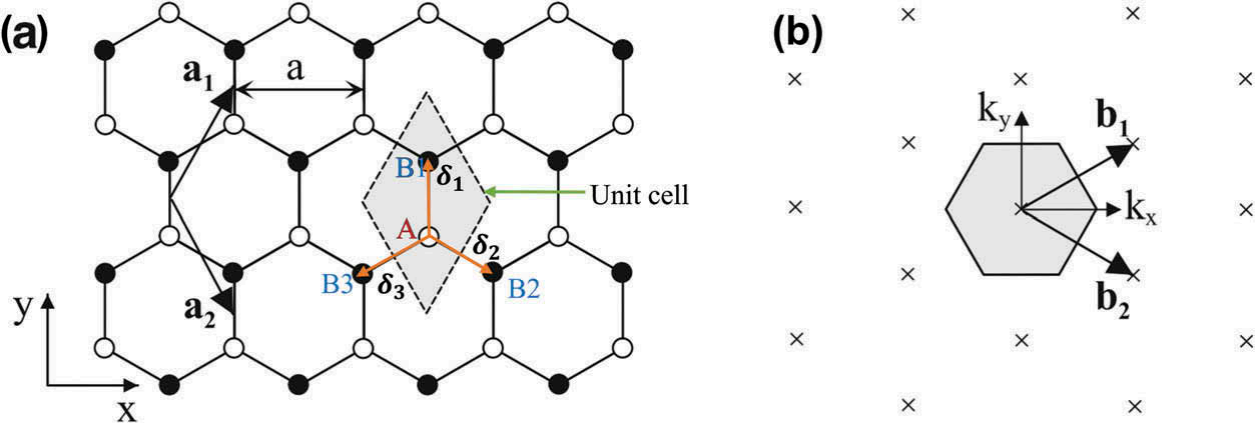
(a) Honeycomb lattice of monolayer graphene, where white (black) circles indicate carbon atoms on A (B) sites, and (b) the reciprocal lattice of monolayer graphene, where the shaded hexagon is the corresponding Brillouin zone [51] (reused with permission from [51] Copyright © 2011, Springer-Verlag Berlin Heidelberg.).

### 2.2. Electronic band structure of graphene

In the hexagonal lattice of monolayer graphene, as seen in Figure 3(a), two primitive lattice vectors are written as:

(1) $${\vec{a}}_{1}=\frac{a}{2}\left(1,\sqrt{3}\right)\;\;\;and\;\;\;{\vec{a}}_{2}=\frac{a}{2}\left(1,-\sqrt{3}\right)$$

where $a=\sqrt{3}a_{0}\approx \sqrt{3}\times 1.42=2.46$ Å is the lattice constant, which is the distance between unit cells. The position vector of atom $B_{l}$, (l= 1, 2, 3) relative to the atom $A_{i}$ is denoted as ${\vec{\delta }}_{l}$, and the three nearest-neighbor vectors in real space are given by

(2) $${\vec{\delta }}_{1}=\left(0,\frac{a}{\sqrt{3}}\right),\;{\vec{\delta }}_{2}=\left(\frac{a}{2},-\frac{a}{2\sqrt{3}}\right)\;\;and\;\;\;{\vec{\delta }}_{3}=\left(-\frac{a}{2},-\frac{a}{2\sqrt{3}}\right)$$

It is noted that $\left|{\vec{\delta }}_{1}\right|=\left|{\vec{\delta }}_{2}\right|=\left|{\vec{\delta }}_{3}\right|=\frac{a}{\sqrt{3}}$ is the spacing between two nearest-neighboring carbon atoms. Figure 3(b) illustrates the reciprocal lattice of monolayer graphene, where the crosses are reciprocal lattice points, and the shaded hexagon is the first Brillouin zone. The primitive reciprocal lattice vectors ${\vec{b}}_{1}$ and ${\vec{b}}_{2}$ satisfy the conditions,

(3) $$\left\{\begin{array}{c} {\vec{a}}_{1}{\vec{b}}_{1}={\vec{a}}_{2}{\vec{b}}_{2}=2\pi \\ {\vec{a}}_{1}{\vec{b}}_{2}={\vec{a}}_{2}{\vec{b}}_{1}=0 \end{array}\right.$$

therefore,

(4) $${\vec{b}}_{1}=\left(\frac{2\pi }{a},\frac{2\pi }{\sqrt{3}a}\right)\;\;and\;\;{\vec{b}}_{2}=\left(\frac{2\pi }{a},-\frac{2\pi }{\sqrt{3}a}\right)$$

Normally, the electronic band structure of graphene can be calculated by using the LCAO method, which is also called tight-binding approach [1,52]. Hence, it is reasonable to start with the Bloch function

(5) $$\Phi_{\alpha }\left(\vec{k},\vec{r}\right)=\frac{1}{\sqrt{N}}\underset{{\vec{R}}_{\alpha }\in G}{\sum }e^{i\vec{k}.{\vec{R}}_{\alpha }}\varphi_{\alpha }\left(\vec{r}-{\vec{R}}_{\alpha }\right)$$

where $\varphi_{\alpha }$ is the wavefunction for the $2p_{z}$ orbitals localized at the position of α-atom, *N* is the number of lattice points, and G denotes a set of lattice vectors. By linearly combining the Bloch function for the two atoms in the unit cell of graphene lattice, we have the electronic eigenfunctions as

(6) $$\Psi_{j}\left(\vec{k},\vec{r}\right)=\overset{2}{\underset{\alpha =1}{\sum }}C_{j\alpha }\left(\vec{k}\right)\Phi_{\alpha }\left(\vec{k},\vec{r}\right)$$

The transfer integral matrix, overlap integral matrix and column vector are given by

(7) $$H=\left(\begin{array}{cc} H_{AA} & H_{AB}\\ H_{BA} & H_{BB} \end{array}\right),\;\;S=\left(\begin{array}{cc} S_{AA} & S_{AB}\\ S_{BA} & S_{BB} \end{array}\right)\;and\;\;C_{j}=\left(\begin{array}{c} C_{jA}\\ C_{jB} \end{array}\right)$$

where the entries $H_{ij}$ and $S_{ij}$ are given by

(8) $$H_{ij}=\langle \Phi_{i}\left|H\right|\Phi_{j}\rangle \;\;and\;\;S_{ij}=\langle \Phi_{i}|\Phi_{j}\rangle$$

The diagonal transfer integral matrix elements $H_{AA}$ can be derived from

(9) $$H_{AA}=\frac{1}{N}\overset{N}{\underset{i=1}{\sum }}\overset{N}{\underset{j=1}{\sum }}e^{i\vec{k}.\left({\vec{R}}_{Aj}-{\vec{R}}_{Ai}\right)}\langle \varphi_{A}\left(\vec{r}-{\vec{R}}_{Ai}\right)\left|H\right|\varphi_{A}\left(\vec{r}-{\vec{R}}_{Aj}\right)\rangle$$

As the dominant contribution comes from *i* = *j*, Equation 9 can be rewritten as

(10) $$\begin{array}{rl} & H_{AA}\approx \frac{1}{N}\sum_{i=1}^{N}\left\langle \varphi_{A}\left(\vec{r}-{\vec{R}}_{Ai}\right)\left|H\right|\varphi_{A}\left(\vec{r}-{\vec{R}}_{Ai}\right)\right\rangle \\ & \;\;\;\;\;\;\;\;\;=\frac{1}{N}\sum_{i=1}^{N}\epsilon_{2p}=\epsilon_{2p} \end{array}$$

where $\epsilon_{2p}$ is the energy of the $2p_{z}$ orbitals of carbon atoms.

Since carbon atoms on sub-lattice B are chemically identical to those on sub-lattice A, we have

(11) $$H_{BB}=H_{AA}\approx \epsilon_{2p}$$

Similarly, the diagonal overlap integrals can be calculated as

(12) $$S_{AA}=S_{BB}\approx \frac{1}{N}\overset{N}{\underset{i=1}{\sum }}\langle \varphi_{A}\left(\vec{r}-{\vec{R}}_{Ai}\right)|\varphi_{A}\left(\vec{r}-{\vec{R}}_{Ai}\right)\rangle \;=1$$

Assuming that the dominate contribution comes from the nearest three neighbors and other contributions are neglected, it is possible to write the off-diagonal transfer integral matrix element as

(13) $$\begin{array}{r} H_{AB}\approx \frac{1}{N}\overset{N}{\underset{i=1}{\sum }}\overset{3}{\underset{l=1}{\sum }}e^{i\vec{k}.\left({\vec{R}}_{Bl}-{\vec{R}}_{Ai}\right)}\\ \left\langle \varphi_{A}\left(\vec{r}-{\vec{R}}_{Ai}\right)\left|H\right|\varphi_{B}\left(\vec{r}-{\vec{R}}_{Bl}\right)\right\rangle \end{array}$$

The value of the matrix element between each nearest-neighboring A and B atoms is the same, so

(14) $$\gamma_{0}=-\langle \varphi_{A}\left(\vec{r}-{\vec{R}}_{Ai}\right)\left|H\right|\varphi_{B}\left(\vec{r}-{\vec{R}}_{Bl}\right)\rangle$$

Then the off-diagonal transfer integral matrix elements can be written as

(15) $$H_{AB}\approx -\frac{\gamma_{0}}{N}\overset{N}{\underset{i=1}{\sum }}\overset{3}{\underset{l=1}{\sum }}e^{i\vec{k}.\left({\vec{R}}_{Bl}-{\vec{R}}_{Ai}\right)}=-\gamma_{0}\overset{3}{\underset{l=1}{\sum }}e^{i\vec{k}.{\vec{\delta }}_{l}}\equiv -\gamma_{0}f\left(\vec{k}\right)$$

(16) $$H_{BA}\approx -\gamma_{0}f^{*}\left(\vec{k}\right)$$

with

(17) $${\vec{\delta }}_{l}={\vec{R}}_{Bl}-{\vec{R}}_{Ai}$$

The function $f\left(\vec{k}\right)$ describing nearest-neighbor hopping can be evaluated as

(18) $$f\left(\vec{k}\right)=\overset{3}{\underset{l=1}{\sum }}e^{i\vec{k}.{\vec{\delta }}_{l}}=e^{i{\vec{k}}_{y}a/\sqrt{3}}+2e^{-i{\vec{k}}_{y}a/2\sqrt{3}}cos\left({\vec{k}}_{x}a/2\right)$$

in a similar fashion

(19) $$S_{AB}\approx -s_{0}f\left(\vec{k}\right)\;\;\;and\;\;\;S_{BA}\approx -s_{0}f^{*}\left(\vec{k}\right)$$

with

(20) $$s_{0}=-\langle \varphi_{A}\left(\vec{r}-{\vec{R}}_{Ai}\right)\left|H\right|\varphi_{B}\left(\vec{r}-{\vec{R}}_{Bl}\right)\rangle$$

Finally, the transfer and overlap integral matrices are obtained

(21) $$H=\left(\begin{array}{cc} \epsilon_{2p} & -\gamma_{0}f\left(\vec{k}\right)\\ -\gamma_{0}f^{*}\left(\vec{k}\right) & \epsilon_{2p} \end{array}\right)$$

(22) $$S=\left(\begin{array}{cc} 1 & -s_{0}f\left(\vec{k}\right)\\ -s_{0}f^{*}\left(\vec{k}\right) & 1 \end{array}\right)$$

The eigenvalues $E_{j}$ (*j* = 1, 2) can be written as

(23) $$E_{j}\left(\vec{k}\right)=\frac{\langle \Phi_{j}\left|H\right|\Phi_{j}\rangle }{\langle \Phi_{j}|\Phi_{j}\rangle }$$

Substituting the expansion in terms of Bloch functions

(24) $$E_{j}\left(\vec{k}\right)=\frac{\sum_{i,l}^{N}C_{ji}^{*}C_{jl}\Phi_{i}\left|H\right|\Phi_{l}}{\sum_{i,l}^{N}C_{ji}^{*}C_{jl}\Phi_{i}|\Phi_{l}}=\frac{\sum_{i,l}^{N}C_{ji}^{*}C_{jl}H_{il}}{\sum_{i,l}^{N}C_{ji}^{*}C_{jl}S_{il}}$$

Minimizing energy with respect to variations of $C_{jm}^{*}$

(25) $$\frac{\partial E_{j}}{\partial C_{jm}^{*}}=0\Rightarrow \overset{2}{\underset{l=1}{\sum }}H_{ml}C_{jl}=E_{j}\overset{2}{\underset{l=1}{\sum }}S_{ml}C_{jl}$$

Written as a matrix equation:

(26) $$HC_{j}=E_{j}SC_{j}\Rightarrow \left(\begin{array}{cc} H_{AA} & H_{AB}\\ H_{BA} & H_{BB} \end{array}\right)\left(\begin{array}{c} C_{jA}\\ C_{jB} \end{array}\right)=E_{j}\left(\begin{array}{cc} S_{AA} & S_{AB}\\ S_{BA} & S_{BB} \end{array}\right)\left(\begin{array}{c} C_{jA}\\ C_{jB} \end{array}\right)$$

The eigenenergies for graphene is then given by

(27) $$det\left[H-E_{j}S\right]=det\left[\begin{array}{cc} \epsilon_{2p}-E_{j} & -\left(\gamma_{0}+E_{j}s_{0}\right)f\left(\vec{k}\right)\\ -\left(\gamma_{0}+E_{j}s_{0}\right)f^{*}\left(\vec{k}\right) & \epsilon_{2p}-E_{j} \end{array}\right]=0$$

By solving this secular equation, the expression for dispersion relation is derived as

(28) $$E_{j}{\left(\vec{k}\right)}_{\lambda }=\frac{\epsilon_{2p}+\lambda \gamma_{0}\left|f\left(\vec{k}\right)\right|}{1-\lambda s_{0}\left|f\left(\vec{k}\right)\right|}$$

where λ=±1 represent the conduction and valence bands respectively. The three parameters $\epsilon_{2p}$, $\gamma_{0}$ and $s_{0}$ can be found by comparison of tight-binding model with fitting experiments, or *ab initio* (from first principles) density functional theory (DFT) [52]. Though it is Wallace who first employed tight-binding model to describe the band structure of graphene. The other nowadays better-known tight binding approximation was given by Saito et al. [53] who considered the nonfinite overlap between the basic functions, but includes only interactions between nearest neighbors within the hexagonal lattice.

**Figure 4.**
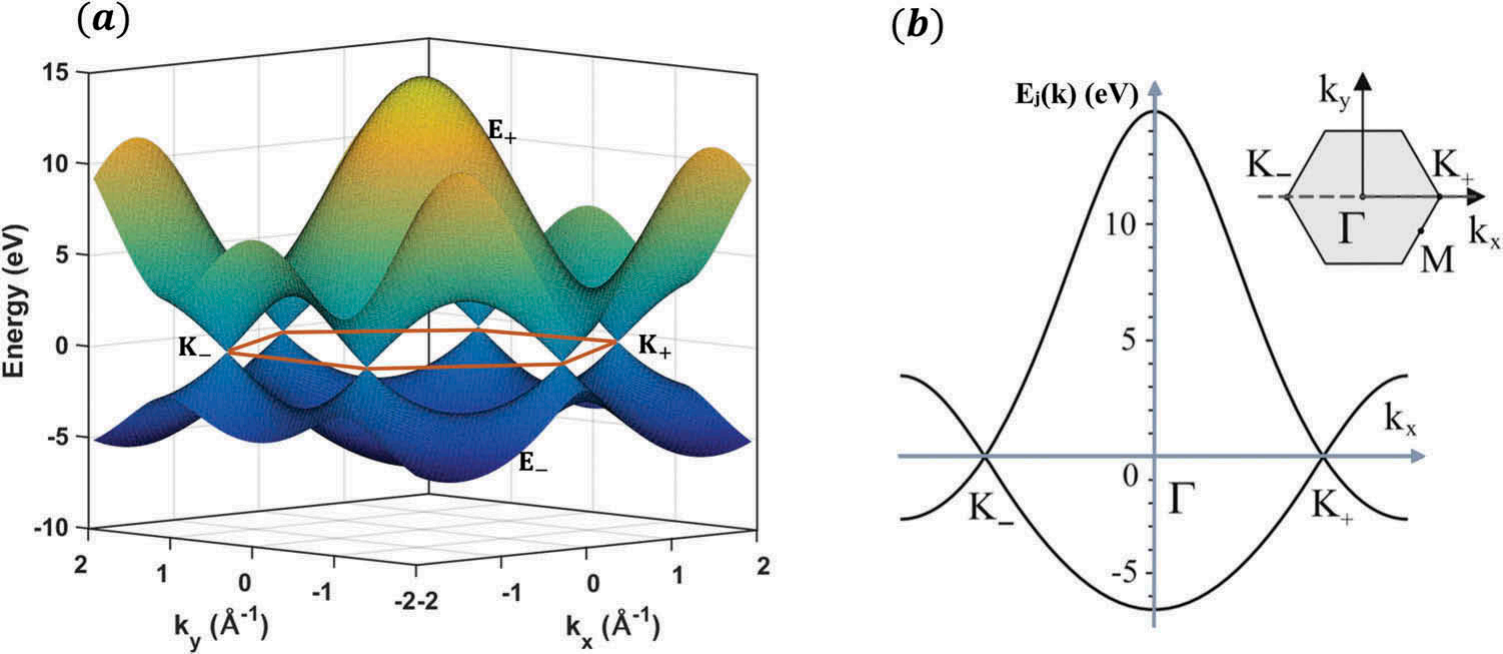
(a) Band structure of graphene calculated with a tight-binding method with $\epsilon_{2p}=0\;eV$, $\gamma_{0}=3.033\;eV$ and $s_{0}=0.129\;eV$. (b) Cross-section through the band structure, where the energy bands are plotted as a function of wave vector component $k_{x}$ along the line $k_{y}=0$.

In a review by Saito et al. [53], the values of $\epsilon_{2p}$, $\gamma_{0}$ and $s_{0}$ are suggested to be 0 eV, 3.033 eV and 0.129 eV. Here, $\epsilon_{2p}=0$ means that the energy of the 2$p_{z}$ orbital is set to be equal to zero. The simulated band structure of graphene plotted in Figure 4(a) is consequently obtained by inputting these three values into the expression 28. Due to considerations of symmetry, the hopping of electrons between the two equivalent carbon triangular sub-lattices in the crystal structure of monolayer graphene leads to the formation of two energy bands (i.e. the upper conduction band and the lower valence band), which intersect at points where $E_{j}\left(\vec{k}\right)$ is identically zero. Furthermore, the Fermi level is located at these points which are also named Dirac points.

A particular line scan of the band structure is shown in Figure 4(b), where the energy bands are plotted as a function of wave vector component $k_{x}$ along the line $k_{y}=0$. In the inserted graph, the center of the Brillouin zone is labeled Γ, while two corners are labeled $K_{+}$ and $K_{-}$ separately. The dispersion near point $K_{+}$($K_{-}$) is linear and can be described by a Dirac-like Hamiltonian [54–56]

(29) $${\hat{H}}_{0}=-i\hbar \nu_{F}\sigma \nabla$$

where ℏ is the reduced Planck constant, $\nu_{F}\approx 10^{6}m/s$ the Fermi velocity, and $\sigma =\left(\sigma_{x},\sigma_{y}\right)$ the Pauli matrices. The asymmetry between the conduction band ($E_{+}$) and the valence band ($E_{-}$), which is especially pronounced in the vicinity of the Γ point, which is attributed to the non-zero overlap parameter $s_{0}$. However, the electronic band structure of graphene can be simply altered by applying electric field [6,57–61] or providing substrates [62,63], and precisely engineered by introducing disorders into the hexagonal lattice [64–68], which will be discussed in detail in later sections.

### 2.3. Edge orientations in graphene

The chemical reactivity of monolayer graphene sheet at edges is at least twice that at basal planes, as suggested by spectroscopic tests and the electron transfer theory [69]. It is also evidenced from STM analysis that edges of graphene can exhibit higher electronic density of states (DOS) near Fermi level than the basal planes [70]. The edge configurations locally determine the distribution of electrons [71], and thus the selection of crystallographic orientation of graphene is of crucial importance for controlling its electronic properties in localized states. Zigzag and armchair are two main types of edges along the crystallographic directions in graphene. In recent years, extensive studies were carried out to investigate the relative stability of these two edge orientations and the edge orientation-dependent physics for mechanically exfoliated monolayer graphene flakes [72–74], epitaxial graphene islands [75–77], CVD derived graphene grains [78], and graphene nanoribbons (GNRs) [79–81].

**Figure 5.**
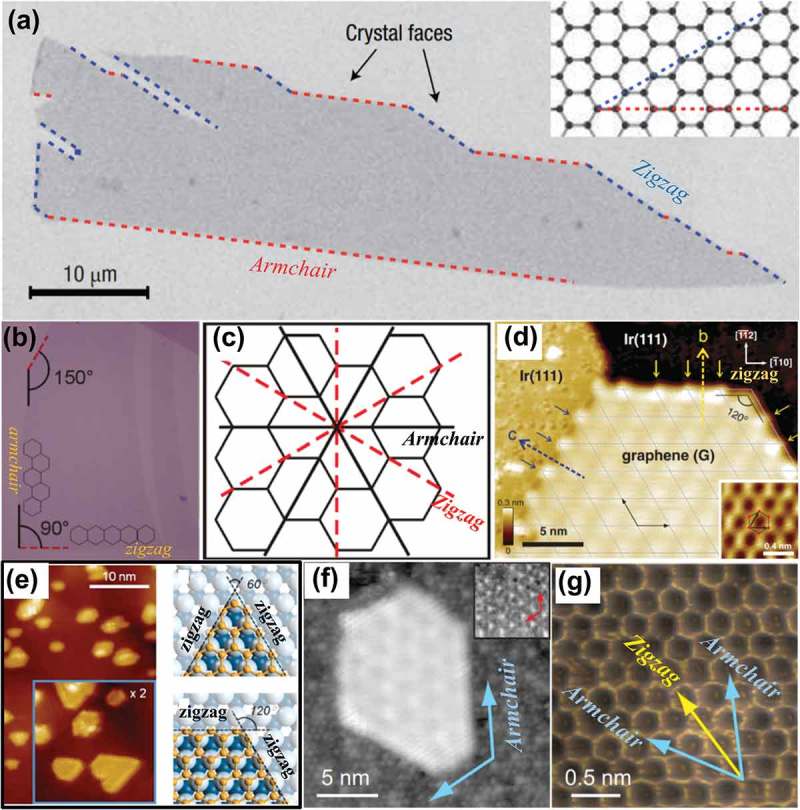
(a) Scanning electron microscopy (SEM) image of a relatively large graphene crystal, which shows that most of the crystal’s faces are zigzag and armchair edges, as indicated by blue and red lines and illustrated in the inset [7]. (b) A typical graphene flake obtained by micromechanical cleavage [72]. (c) Sketch of the honeycomb crystal lattice of graphene. Two distinct crystallographic orientations of a graphene crystal, rotated against each other in multiples of 30°, are indicated as armchair type (solid lines) and zigzag type (dashed lines) [72]. (d) CC-STM image of edges of graphene on Ir(111), with crystallographic directions of the Ir substrate denoted at the top-right side [89]. (e) STM image of graphene structures on Co substrate, and schematic of triangular and hexagonal corners, respectively, for zigzag-edged graphene structures on Co(0001) [90]. (f) 20 × 20 nm^2^ STM image and (g) 2.5 × 2.5 nm^2^ rendered STM topography of graphene island on 6H-SiC(0001) substrate. Overlaid on this image are the two lowest energy edge directions: zigzag (yellow arrow) and armchair (blue arrows) [75] (reused with permissions from [7] Copyright © 2007, Springer Nature, [72] Rights managed by AIP Publishing, [89] Copyright © 2012 American Physical Society,  [90] Copyright © 2014, American Chemical Society, and [75] Copyright ©2010 American Physical Society.).

In mechanical exfoliation of graphene flakes, the breaking is suggested to occur along the principle crystallographic directions [7,82]. Moreover, due to the hexagonal symmetry of graphene crystal (see Figure 5(c)), the resulting edges of graphene flakes are expected to be terminated with either armchair or zigzag edges. As a consequence, the edges of mechanically exfoliated graphene flakes are mostly straight, and the angles between adjacent edges are often a multiple of 30° [7,72], as seen in Figure 5(a,b). On the other hand, zigzag directions appear to be more favorable for edges formed by certain etching reactions [83,84], or in holes created by electron beam irradiation in graphene [85,86]. The edge orientations in graphene flakes can be determined by using high-resolution STM which is capable of providing atomic resolution images of the graphene crystal lattice for unambiguous identification of armchair or zigzag edges [72]. Edge orientations of monolayer graphene can also be identified by G mode in Raman spectroscopy [73], as G modes at zigzag- or armchair-dominated edges of monolayer graphene exhibit different polar behaviors. Xu et al [74] employed polarized Raman spectroscopy to investigate the thermal stability and dynamics of graphene edges, and they found that both zigzag and armchair edges were unstable and underwent modifications even at 200 °C. Hyun et al. [87] further suggested that the edges of graphene flake have predominantly zigzag terminations below 400 °C, while the edges would be dominated by armchair and reconstructed zigzag edges above an annealing temperature of above 600 °C. Recent studies on the processes and mechanisms which drive the chemical functionalization of graphene edges are reviewed by Bellunato et al. [71].

Graphene islands are of great scientific interest due to their small sizes which are suspected to yield novel electronic properties (e.g. quantum confinement [88]). The edge orientations of epitaxial graphene islands are found to be strongly influenced by the underlying substrates. For example, graphenenano-islands that are epitaxially grown on monocrystalline metal surfaces (i.e. Ir(111) [76,89], Co(0001) [77,90] and Pt(111) [91]) have mostly zigzag terminations, whereas those grown on 6H-SiC(0001) are dominated by armchair edges [75]. The constant-current STM (CC-STM) image of edges of graphene on Ir(111) can be seen in Figure 5(d), where the gray mesh indicates the moire superstructure on graphene, and the blue and yellow arrows represent the zigzag graphene edges on substrate surface and at substrate step edges respectively. It is evidenced from low-temperature transmission electron microscopy (TEM) measurements that graphene structures exhibit an on-top registry with respect to the Co(0001) surface [77]. A detailed analysis of the on-top registry reveals that the straight edges of graphene on clean Co surface are mainly terminated in zigzag directions. With fixed edge chirality, commensurate graphene can form islands on Co(0001) substrate with either triangular or hexagonal corners, as seen in Figure 5(e) [90]. In the first case, the peripheral atoms maintain the same registry, whereas, in the second case, adjacent edges exhibit opposite configurations. From Figure 5(f), the edges of the graphene island which are highlighted by overlaying two blue arrows are in alignment with the lattice vector directions of underlying SiC substrate (see the red arrows in the inset) [75]. An atomic-resolution STM image of the step edges of this graphene island is provided in Figure 5(g), where the blue and yellow arrows indicate armchair and zigzag directions respectively. By comparing the arrows in Figure 5(f,g), the graphene island is ascertained to be entirely surrounded by armchair edges.

**Figure 6.**
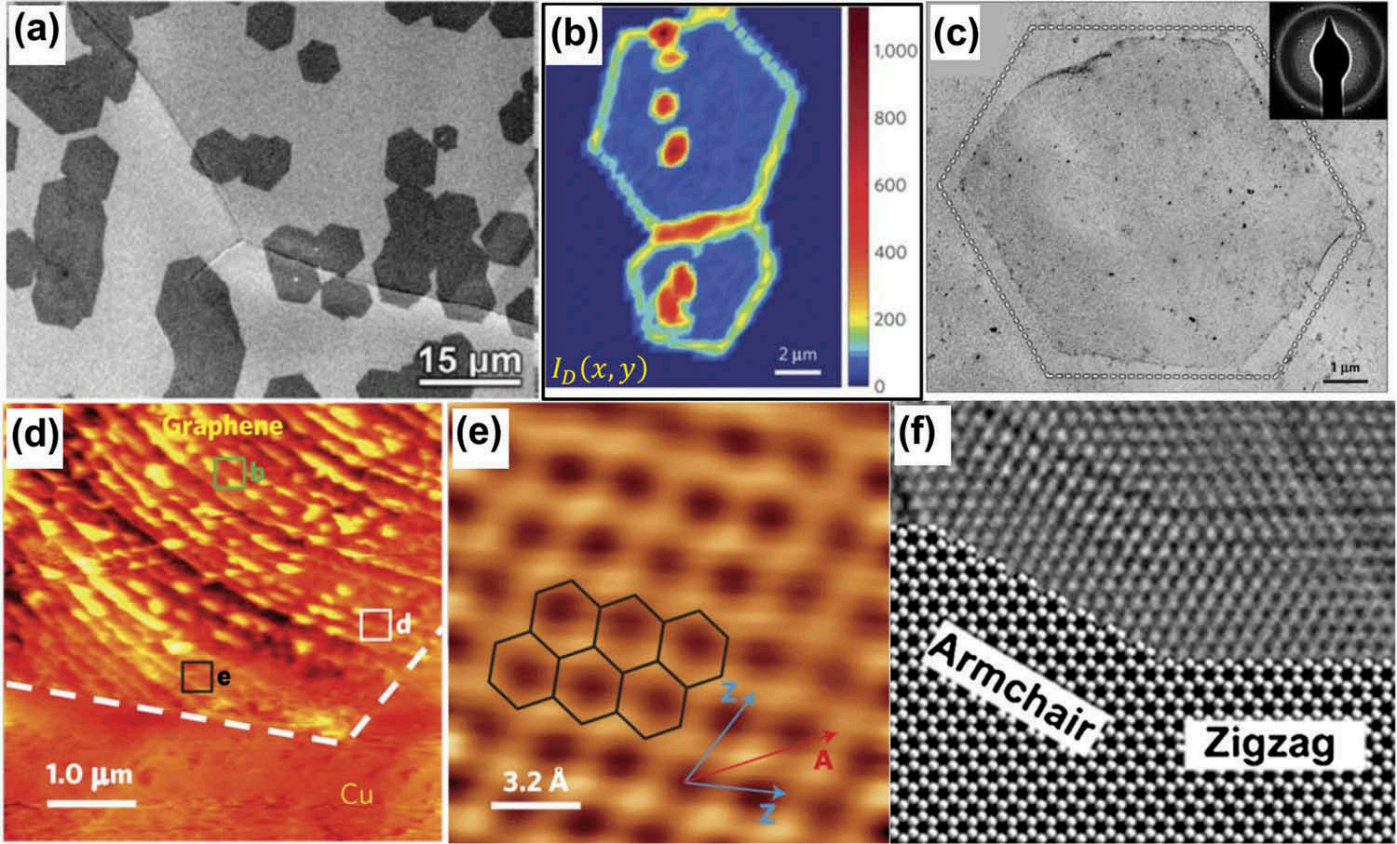
(a) SEM image of an annealed Cu foil taken out of the furnace after graphene growth [78]. (b) Intensity map of D band for two coalesced graphene grains [78]. (c) A montage of bright field TEM images (80 kV) spliced together to show an example of a hexagonally shaped graphene grain, with its characteristic SAED pattern in the inset [78]. (d) STM topography image taken near a corner of a graphene grain on Cu [78]. (e) Atomic-resolution STM image taken from the area which is marked by a black square in Figure 6(d). (Z: zigzag; A: armchair) [78]. (f) A modified HRTEM image of overlapping zigzag-armchair edges; the HRTEM image is originally from Ref [79], and the modified version is from Ref [111] (reused with permissions from [78] Copyright © 2011, Springer Nature, [79] Copyright © 2009, American Association for the Advancement of Science, and [111] Copyright © 2010 Elsevier Ltd.).

In the CVD growth of polycrystalline graphene, graphene boundaries (GBs) are formed by coalescence of the graphene grains that initially nucleate from random and uncontrollable locations. These GBs can impede electron transport [92] and degrade the mechanical properties of the resulting films [93]. Figure 6(a) indicates the formation of isolated grains and merged grains at the early stage of ambient CVD growth of graphene on Cu substrates [78]. As shown in Figure 6(b), Raman mapping of the D peak intensity can provide a clear identification of the locations of grain boundaries between two coalesced grains, which cannot be visualized by simply using SEM or atomic force microscopy (AFM). The few isolated spots displaying relatively large D peak intensities are suggested to be the nucleation centers where the CVD-growth of graphene is initiated [78]. Pronounced D peak intensities were also observed at the grain edges, consistent with previous Raman studies [94,95]. The edge orientations of graphene grains can be determined by comparing their TEM images in real space with the corresponding selected area electron diffraction (SAED) patterns (see the supplementary information in Ref [78].). By using this identification method, most of the grain edges were found to be approximately aligned with zigzag directions. The bright filed TEM image of a typical hexagonally shaped graphene grain and its characteristic SAED pattern (inset) are given in Figure 6(c), where the dashed lines correspond to the zigzag directions. Figure 6(d) shows a large-scan-area STM topography image of a graphene grain near a hexagonal corner, and the atomic-resolution STM image in Figure 6(e) is taken from the area marked by a black square in Figure 6(d). By comparing the grain edges (white dashed lines) in Figure 6(d) with the crystal directions (zigzag versus armchair) in Figure 6(e), it is confirmed that the edges of CVD derived hexagonal grains are mostly bounded by zigzag terminations.

With decreasing size, the surface free energy of a material increases, and its properties start depending on the surface interactions. This size effect is more significant for nanomaterials, with an average size of 1–100 nm, for most of their atoms are exposed to the surface and interact with the surroundings. For the same reason, the electronic structure of GNRs that have ultrathin width of less than 10 nm are quite sensitive to their edge orientations. The approaches for synthesis of GNRs include epitaxial growth on templated silicon carbide (SiC) substrates [96], CVD growth on structured substrates (e.g. steps [97], twins [98], and trenches [99]), and directly partial growth on germanium(001) [100]. The lattice of GNR shows both zigzag and armchair edge orientations which can be observed by using AFM [101] and STM [79,100]. Figure 6(f) illustrates a modified high-resolution TEM (HRTEM) image of overlapping zigzag-armchair edges. Normally, GNRs with a higher fraction of zigzag edges exhibit a smaller energy gap than a predominantly armchair-edge ribbon of similar width [80]. On the basis of tight binding theory, the zigzag GNRs are always metallic, while the armchair GNRs exhibit either metallic or semiconducting behaviors, depending on their width. For instance, DFT shows that the energy band gaps of semiconducting armchair GNRs increase as their width decreases [81]. This property of armchair GNRs is also evidenced from experiments [102]. Therefore, the electronic structure of GNRs can be engineered by modification of local structure of edge [103], altering GNR configuration (e.g. width and crystallographic orientation) [104,105] as well as electron–electron and spin–orbit interactions [59,106]. More detailed information about the design of electronic properties of GNRs was given by Dutta and Pati [107] and Yazyev et al. [108].

### 2.4. Number of graphene layers

The term graphene theoretically refers to monolayer graphene [109], and sometimes also includes bilayer graphene, as both of them are semimetals with no overlap between the valence and conduction bands [7]. The electronic structure of few-layer graphene (FLG, number of layers from 3 to < 10), is more complex because of the appearance of charge carriers. It has been shown that the electronic structure of graphene rapidly evolves with the number of layers, approaching the 3D limit of graphite at 10 layers [110].

**Figure 7.**
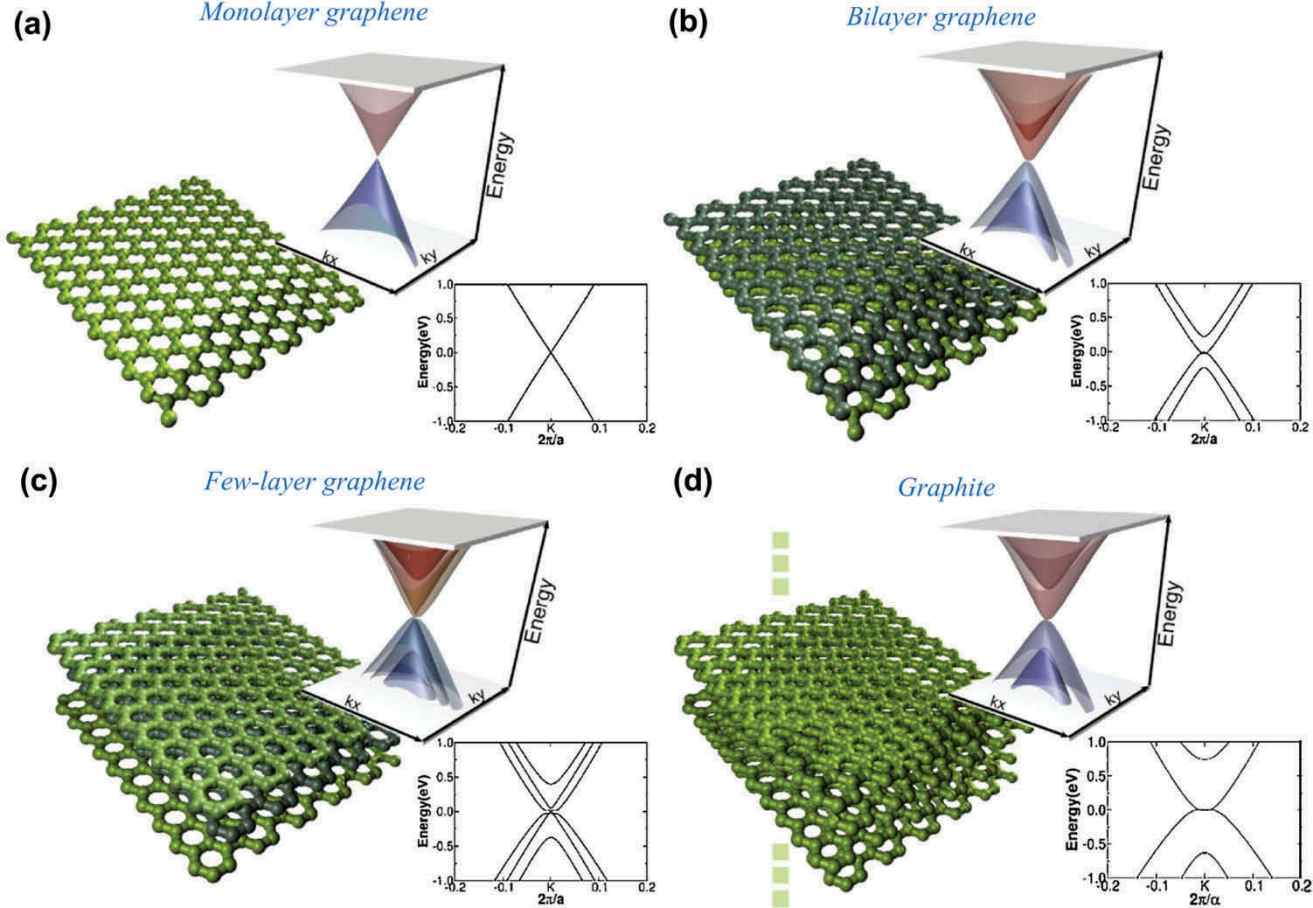
Low energy DFT 3D band structure and its projection on $k_{x}$ component close to K point for (a) monolayer graphene, (b) *AB*-stacked bilayer graphene, (c) ABA-stacked trilayer graphene and (d) bulk graphite. The Fermi level has been set at zero in all cases [111] (reused with permission from [111] Copyright © 2010 Elsevier Ltd.).

Figure 7 illustrates the low-energy DFT 3D band structure and its projection on $k_{x}$ component close to K point for monolayer, bilayer, and trilayer graphenes and bulk graphite [111]. In the energy spectrum of monolayer graphene, the conduction band and valence band touch at Dirac points, and the electron dispersion near these points is linear. In monolayer graphene, there is no underneath carbon atom for the $2p_{z}$ orbital to interact with, whereas this possibility exists in bilayer graphene, which enables the formation of a zero-energy band. Owing to the presence of massive chiral quasiparticles with parabolic dispersion at low energy [112], the integer quantum Hall effect in bilayer graphene [113] can be even more unusual than that in monolayer graphene. Figure 7(b) shows the four parabolic bands, as the (AB-stacked) bilayer graphene has four atoms in the unit cell. The band structure of bilayer graphene can be tuned by applying an electric field [114,115], providing appropriate substrates [116] or chemical modulations [117,118], which is expected to attract interests in nanoelectronic and nanophotonic applications [119]. From Figure 7(c), the band structure of (ABA-stacked) trilayer graphene seems to be a combination of those of monolayer and (AB-stacked) bilayer. However, trilayer graphene is actually a semimetal with a conductivity that increases with increasing electric field. This behavior significantly differs from that of monolayer and bilayer graphene, which is originated from the presence of a finite overlap between valence and conduction band [120]. Moreover, as effective mass of graphene increases with the increasing layer thickness, trilayer graphene exhibits lower mobility than those of monolayer and bilayer [121]. In general, the low-energy spectrum of FLG with odd number of layers is a combination of one massless Dirac mode and N−1 massive Dirac modes per spin and valley, whereas all N modes are massive at low-energies for even number of layers. Therefore, for FLG with N layers (AB stacking), there will be $\frac{N-1}{2}$ electronlike and $\frac{N-1}{2}$ holelike parabolic bands and an additional linear energy band (Dirac fermions) around K point [122] if N is odd; Otherwise, there will be only $\frac{N}{2}$ electronlike and $\frac{N}{2}$ holelike parabolic bands around K point. Because of a significant overlap between the conduction and valence bands, FLG thicker than five layers shows a semi-metallic band structure with parabolic-like bands, which is highly similar to that of bulk graphite, as seen in Figure 7(d).

Not surprisingly, properties of bilayer graphene are quite similar to those of monolayer graphene. These properties include excellent room temperature electrical conductivity with mobility of up to 40,000 cm^2^ V^−1^ s^−1^ [123], high room temperature thermal conductivity of about 2800 W m^−1^ K^−1^ [124,125], outstanding mechanical stiffness and strength with a Young’s modulus of about 0.8 TPa [126,127], fine transparency with white light transmittance of about 95% [128], and impermeability to gases [129]. Zhang and Gu [130] carried out molecular dynamics (MD) simulations on multi-layer graphene with layer number varying from one to seven, and found that the mechanical properties (i.e. Young’s modulus, fracture stress and fracture strain) of graphene were sensitive to the temperature changes rather than the layer numbers and isotope substitutions. Lee et al. [131] conducted tensile and friction testing of graphene sheet (with 1–4 layers) by using AFM tips. The experimental results indicated that little difference was found in the intrinsic stiffness and strength of sample with 1–3 atomic layers, while the friction force between AFM tip and graphene sheet decreases as the layer number increases from 1 to 4. In the calculation model established by Xiao et al. [132], the electrical conductivity of few-layer graphene with layer number of 2–9 reduced as the thickness increased, and the reduction of conductivity was mainly caused by the inhibited carrier mobility. Further, the conductivity decreased rapidly with increasing thickness when the number of graphene layers ranged from 2 to 13, but decreased slowly and thereafter remained constant for layer numbers up to 165. These numerical simulation results were in agreement of the published experimental results [133,134]. More information about the properties of monolayer, bilayer and few-layer graphene can be found in Refs [119,135–139].

**Figure 8.**
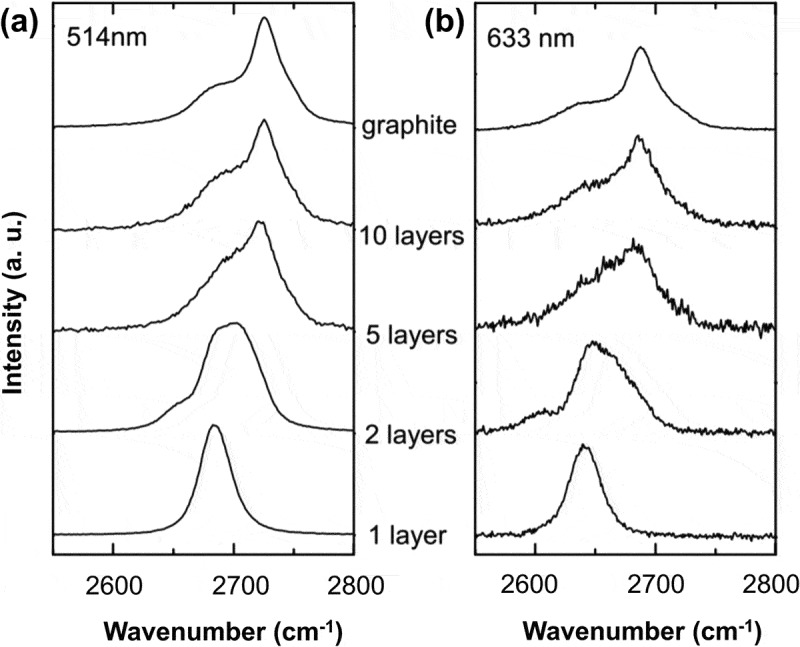
Evolution of the (a) 514 nm and (b) 633 nm Raman spectra near the 2D peak with the number of graphene layers [140] (reused with permission from [140] Copyright © 2006 American Physical Society.).

The 514 nm and 633 nm Raman spectra around the wavenumber of 2700 $cm^{-1}$ for graphenes with various numbers of layers are compared in Figure 8. By examination of the location and shape of defect-activated D peak and the most two prominent features (G and G’ peaks) in the Raman spectra of graphenes with less than five layers, the number of graphene layers can be effectively identified without ambiguity [140,141]. However, the Raman spectrum of FLG thicker than five layers can be hardly distinguished from that of bulk graphite, as the stepwise broadened 2D band approaching that of bulk graphite due to continuous splitting of valence and conduction bands. The number of graphene layers can also be determined by SEM [142], Auger electron spectroscopy (AES) [143], nanoindentation [144], optical reflection microscopy [145] and surface plasmon resonance (SPR) [146,147].

Recently, multiple attempts have been made to precisely control the number of layers for the purpose of tuning the electronic band structure of graphene [111]. For e.g. Lin et al. [148] used a picosecond laser to remove graphene layers from multi-layered graphene films. As both the lateral size and the crystallinity of the starting graphite influenced the number of graphene layers obtained by chemical exfoliation, Wu et al. [149] successfully tuned the number of graphene layers by selecting appropriate starting materials from artificial graphite, flake graphite powder, Kish graphite, and natural flake graphite. In that study, the number of graphene layers was measured by AFM.

### 2.5. Stacking arrangements of graphene layers

**Figure 9.**
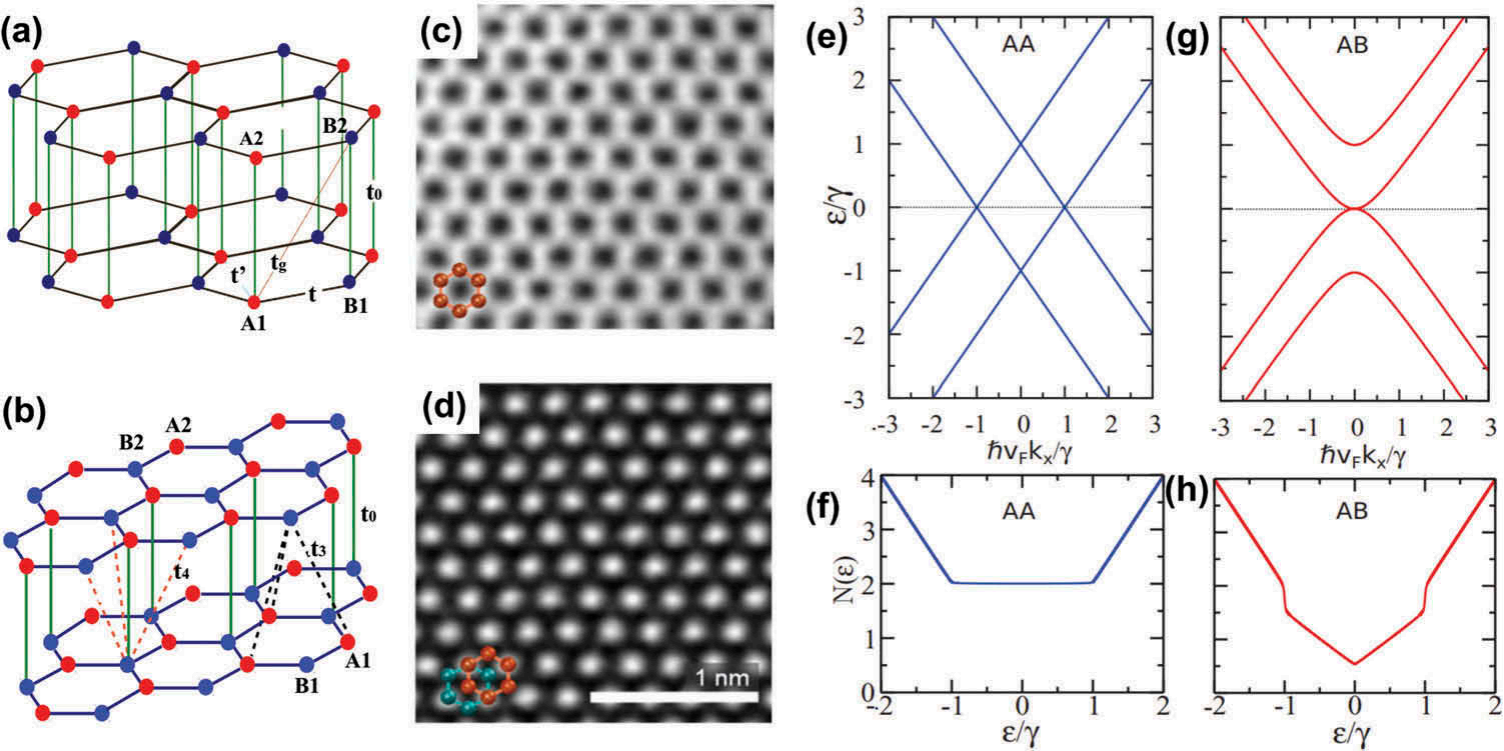
Stacking arrangements and hopping parameters for (a) AA-stacked and (b) AB-stacked bilayer graphene [166]. Hopping integrals ***t*** and ***t’*** correspond to the in-plane nearest-neighboring and next-nearest-neighboring hopping respectively, and parameter $t_{0}$ is associated with the main interlayer hopping in bilayer. Atomic-resolution STEM images of (c) AA- and (d) AB-stacked bilayer graphenes [156]. Hexagonal ring in the first (second) or bottom (top) layer is marked with green (orange) color. (e) Low energy dispersion and (f) low energy DOS for a bilayer graphene with AA stacking. (g) Low-energy dispersion and (h) low-energy DOS for a bilayer graphene with AB stacking [157] (reused with permissions from [166] © 2016 Elsevier B.V., and [157] Copyright © 2012 American Physical Society.).

The crystallographic stacking of graphene sheets provides additional degrees of freedom, thus leading to countless stacking sequences [150,151]. The different stacking orders of the honeycomb network of planar structures strongly influence the interlayer screening [152], band structure [153,154], and spin–orbit coupling [155] of the resulting graphene films. The stacking arrangements of bilayer graphenes can be either AA or AB, which are shown in Figure 9(a,b). For AA stacking, each carbon atoms in the second layer directly aligned on the top of another atom in the first layer, while in bilayer graphene with Bernal or *AB* stacking, a set of atoms in the second layer sit over the empty centers of hexagons in the first layer. As seen in the scanning transmission electron microscopy (STEM) image of AA-stacked bilayer graphene (see Figure 9(c)) [156], all atomic sites are visible in a hexagonal array and show similar brightness. Whereas in Bernal-stacked bilayer, bright spots having hexagonal symmetry and a spacing of 0.25 nm (close to $\sqrt{3}a_{0}$) are observed in Figure 9(d). These spots, which correspond to the sites where two atoms are stacked on top of one another, are threefold to fourfold brighter than that of individual atoms because of the coherent scattering.

Bilayer graphene has not only in-plane hopping (e.g. nearest neighboring hopping and next nearest neighboring) in the same layer, just like that in monolayer graphene, but also vertical hopping which is dependent on the stacking order. In the case of AA-stacked bilayer graphene, vertical hopping mainly occurs between $A_{1}$–$A_{2}$ sites and $B_{1}$–$B_{2}$ sites, while in *AB*-stacked bilayer, the vertical hopping can be very complex: besides the hopping between the dimer sites $t_{0}$, it is possible to introduce the hopping $t_{3}$ from a non-dimer site to nearest non-dimer sites in the opposite layer, and hopping $t_{4}$ from a dimer site to nearest non-dimer sites of the opposite layer. Consequently, subtle difference exists in the electronic structure of bilayer graphene with different stacking orders.

The single spin Hamiltonians for AA-stacked and AB-stacked bilayer graphene and their corresponding matrix representations are given in Refs [157,158]. By calculation of the eigenvalues of these two matrices, the band structures of two types of bilayer are obtained and the corresponding low-energy parts are plotted in Figure 9(e,g). Analytic expressions for the total double spin DOS of two bilayers are derived from the sum of two Dirac cone DOS which are shifted relative to each other by 2γ, and two plots of low-energy DOS are then compared in Figure 9(f,h). Because of the differences in electronic structure, AA-stacked and AB-stacked bilayers exhibit different optical conductivity [157] and responses to an external electric field [159]. The energy gap of bilayer graphene can be artificially opened by application of a perpendicular electric field [160], hence a controlled induction of an insulating state in bilayer graphene can be realized by using a dual-gate device [161]. Although AB stacking is energetically the most favorable in bilayer graphene, the non-AB stacking (e.g. AA stacking and AA’ stacking) regions can be stabilized by means of creating stacking boundaries [162] or introducing a minute twist [163]. Moreover, the formation of closed edges between adjacent graphene layers results in that AA stacking is more frequently seen in bilayer graphene so that the local strains can be reduced [164]. Further, extensive theoretical and experimental studies have been carried out to explore the properties of AA-stacked [157,164–166] and Bernal-stacked [139,158,167,168] bilayer graphenes.

**Figure 10.**
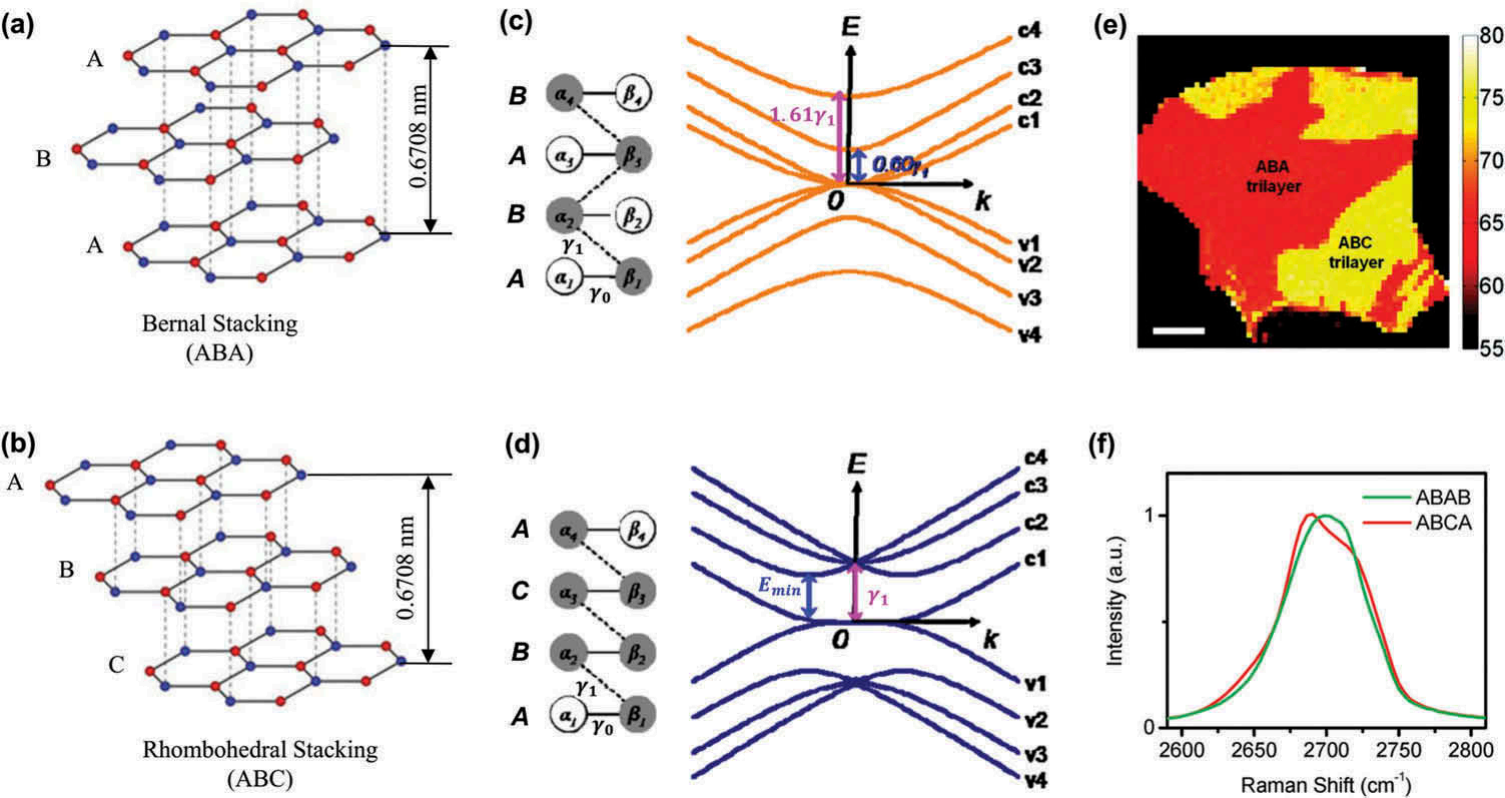
(a) Bernal and (b) rhombohedral stacking arrangements in multilayer graphene. Electronic band structures of (c) Bernal-stacked and (d) rhombohedral stacked tetralayer graphenes [150]. (e)Raman imaging of the distribution of ABA and ABC trilayer graphene domains [174]. (f) Raman 2D-mode spectra for the tetralayer graphene samples of ABAB (green line) and ABCA (red line) stacking orders [174](reused with permissions from [150] Copyright © 2010 American Physical Society, and [174] Copyright © 2011, American Chemical Society.).

The stacking orders can become more complicated for multi-layer graphene. Theoretically, there are two stable stacking arrangements, namely Bernal (in ABA stacking order) and rhombohedral (in ABC stacking sequence), as seen in Figure 10(a,b), and Bernal structures are presumed to be slightly more thermodynamically stable. In both cases, the stacking of graphene layers is attributed to the weak interaction between π bonds in the adjacent basal planes, and these two structural configurations have the same stacking distance of 0.3354 nm between sheets. However, the stacking sequence of FLG is experimentally found to exert pronounced influence on the electronic band structure [150], which are compared in Figure 10(c,d), and thus the electrical properties of FLG with different stacking sequences are expected to be distinct to some extent. For example, the Bernal trilayer is viewed as a semimetal with electrically tunable band overlap [120,169–171], while the rhombohedral trilayer is predicted to be a semiconductor with a band gap that can be electrically tuned [153,169,170]. Moreover, the application of a certain electric field can finally open larger band gap in the latter than that in the former [153,172].

The controlled growth of FLG with specified stacking sequences can be performed in the chamber of an environmental scanning electron microscope (ESEM) which enables real-time imaging of the shape and size evolution of graphene islands [173]. Infrared (IR) absorption/reflection spectroscopy can be used to characterize the stacking order in graphene [124], but this technique has a low spatial resolution [150]. In contrast, the stacking structures in tri- and tetralayer graphenes are readily visualized by Raman imaging, owing to the clear associations with the distinctive line shapes and widths in the Raman 2D mode [174] (see Figure 10(e,f)). The stacking order and corresponding number of layers in multi-layer graphene can also be characterized by TEM [175].

## 3. Disorders in graphene structure

An ideal graphene with highly ordered structures exhibits zero band gap [6], high tensile strength [14] and high thermal conductivity [15] at room temperature. Despite such outstanding properties, the potential applications of atom-thick graphene are limited, so it needs to be incorporated with other materials or assembled into nanopapers [176–178], functional thin films [179], fibers [180,181] and coatings [30,182] for meeting various demands in industry. Some properties of graphene might be enhanced when completing the assembling processes, but large disorder is introduced to the graphene crystal as well. Disorders can also be brought into the crystal structure of graphene during the synthesis process. These disorders can be generally categorized as corrugations, topological defects, adatoms, vacancies and sp^3^-defects. Therefore, this part will first introduce the configurations of diverse types of disorders contained in the crystal structure of graphene in detail, followed by a brief discussion about the formation energy and immigration energy of these disorders. After that, the influence of different types of disorders on properties of graphene are analyzed. Lastly, we will introduce the generation of various disorders during graphene preparation procedures, and provide approaches for reducing these disorders, so that near-defect-free samples of monolayer graphene can be obtained.

### 3.1. Corrugations

In the standard harmonic approximation [183], the long-range order of two-dimensional lattices should be destroyed by thermal fluctuation, so a perfectly flat graphene is presumed to be non-existent [3,184]. Moreover, it is experimentally proven [185–187] that ultrathin films with only dozens of layers are thermodynamically unstable unless they inherently constitute a part of three-dimensional structures (e.g. a substrate with a matching lattice). Indeed, the atomically thin film can be significantly stabilized by partially decoupled bending and stretching modes [188]. By using TEM, a monolayer graphene was found to be freely suspended on a microfabricated metallic scaffold in both vacuum and air at room temperature, with random elastic deformations in the third dimension [9]. The asymmetric distribution of carbon–carbon bond lengths resulting from the localized π electrons forces the graphene lattice to become non-planar for minimizing free energy, thus leading to the formation of ripples with heights up to 1 nm. Therefore, the thermal fluctuations induced ripples are intrinsic features on graphene sheets. As observed under STM [189], these spontaneous ripples on the suspended graphene are dynamic. Plus, the density of the ripples is higher near the edges or defects due to the amplified asymmetry of the bond lengths at these regions [188,190]. The amplitude of ripples is limited by the strain energy induced by its perpendicular displacement, and increases with the size of graphene sheet [191]. The spatial distributions of ripples can be derived from the equation [188]:

(30) $$L=4\pi \kappa {\left(\frac{2\pi }{3TB}\right)}^{\frac{1}{2}}$$

where *L* is the distance between two ripples, κ the bending rigidity, T the absolute temperature and B the two-dimensional bulk modulus. The equation indicates that the distance between two ripples is inversely proportional to the square root of the temperature, and approaches infinity at the temperature of 0 K.

**Figure 11.**
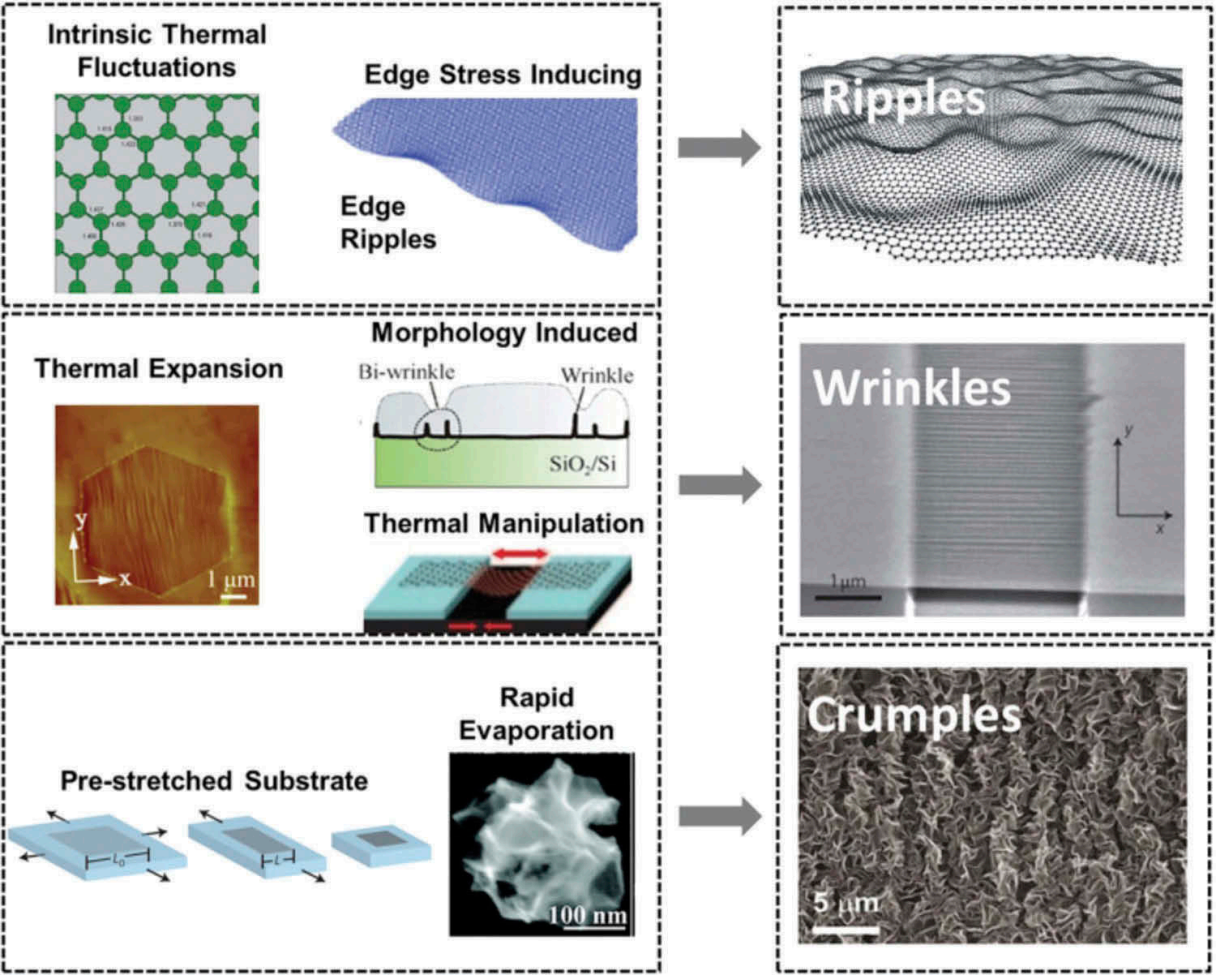
A summary illustration of three types of corrugations (i.e. ripples, wrinkles and crumples) [192]. (reused with permission from [192] © 2015 The Authors. Published by Elsevier Ltd.).

Apart from the intrinsic disorder of ripples, wrinkles and crumples are other two types of corrugations in graphene sheets [192] (see Figure 11). Unlike ripples having a modest aspect ratio of ~ 1 and feature sizes of peaks and valleys below 10 nm, wrinkles exhibit a high aspect ratio of 10, and the width ranges from 1 to 10 nm, the height below 15 nm and the length above 100 nm [193]. Wrinkles normally occur on metallic substrates due to the opposite thermal deformation [194,195] and the defect lines [196] on the substrates. Also, the thickness of growth substrate has an effect on the attributes and density of wrinkles. For instance, as the thickness of nickel substrate increases, the grain size of supported graphene decreases, leading to higher-density and smaller wrinkles on graphene sheet [194]. Wrinkles can also be generated by water drainage between graphene and substrate in the graphene transfer processes. The relation between wavelength (λ) and amplitude (A) of wrinkles on suspended FLG resulted from longitudinal stretching strain [197] can be described by [198]

(31) $$\lambda =\frac{tL}{A}{\left(\frac{8\nu }{3\left(1-\nu^{2}\right)}\right)}^{\frac{1}{2}}$$

where t and *L* are the thickness and width of a graphene thin film respectively, and ν is the Poisson’s ratio which is predicted to be 0.1–0.3 for monolayer graphene [199,200]. However, the relation needs to be revised slightly when in-plane shear dominates the applied stress [198]:

(32) $$\lambda =\frac{tL}{A}{\left(\frac{8}{3\left(1-\nu \right)}\right)}^{\frac{1}{2}}$$

As the values of λ, A, t and L can be measured from AFM images, plotting $\frac{\lambda A}{L}$ versus t allows the determination of the type of applied stress [198]. When using the buckling theory to calculate the wrinkling wavelength of the pre-strained substrate-supported graphene thin film, the substrate is assumed to exhibit stress–strain behavior complying with Hooke’s law but be non-linear at large deformations, so we have:

(33) $$\lambda =2\pi t{\left(\frac{E}{12\Lambda \mu_{s}\left(1-\nu^{2}\right)}\right)}^{\frac{1}{3}}$$

where t is the thickness of the graphene film, E the Young’s modulus of graphene, $\mu_{s}$ the shear modulus of substrate, ν the Poisson’s ratio and $\Lambda =\left(1+{\left(1+\varepsilon_{pre}\right)}^{3}\right)/\left(2\left(1+\varepsilon_{pre}\right)\right)$, for $\varepsilon_{pre}=\left(L_{pre}-L_{0}\right)/L_{0}$, where $L_{pre}$ and $L_{0}$ are pre-strain and initial length of the substrate respectively [201].

Crumples are dense deformations that are generated by rapid evaporation, usually occurring isotropically in two or three dimensions [189,201]. For example, submicrometer-size ball-like crumpled graphene structures can be produced by isotropic compression and thermal reduction of GO [202]. Ma et al. [203] also reported the fabrication of few hundred nanometer-size crumped graphene ball by rapid drying of GO. Unlike wrinkled graphene that experiences deformation by uniaxial confining forces, crumpled graphene undergoes multidirectional compression. The increasing lateral compression force makes the graphene thin film to transform from flat to cone and finally to crumple ball [204]. When the compression force is larger than the crumpling threshold state ($F_{c}$), the radius of graphene sheet decreases to 63% of its initial value, and is nearly independent of force. In this case, the crumpled graphene sheet is expected to follow a power-law behavior, and has a scaling form [204] as:

(34) $$R_{f}=R_{0}C{\left(\frac{E_{0}R_{0}^{2}}{\kappa }\right)}^{\beta }{\left(\frac{\kappa }{FR_{0}}\right)}^{\alpha }$$

where $R_{f}$ and $R_{0}$ are sheet radius under force and the initial sheet radius respectively, $E_{0}$ the 2D Young’s modulus, κ the bending rigidity, F the compression force, and C, β, α the scaling parameters.

### 3.2. Topological defects

**Figure 12.**
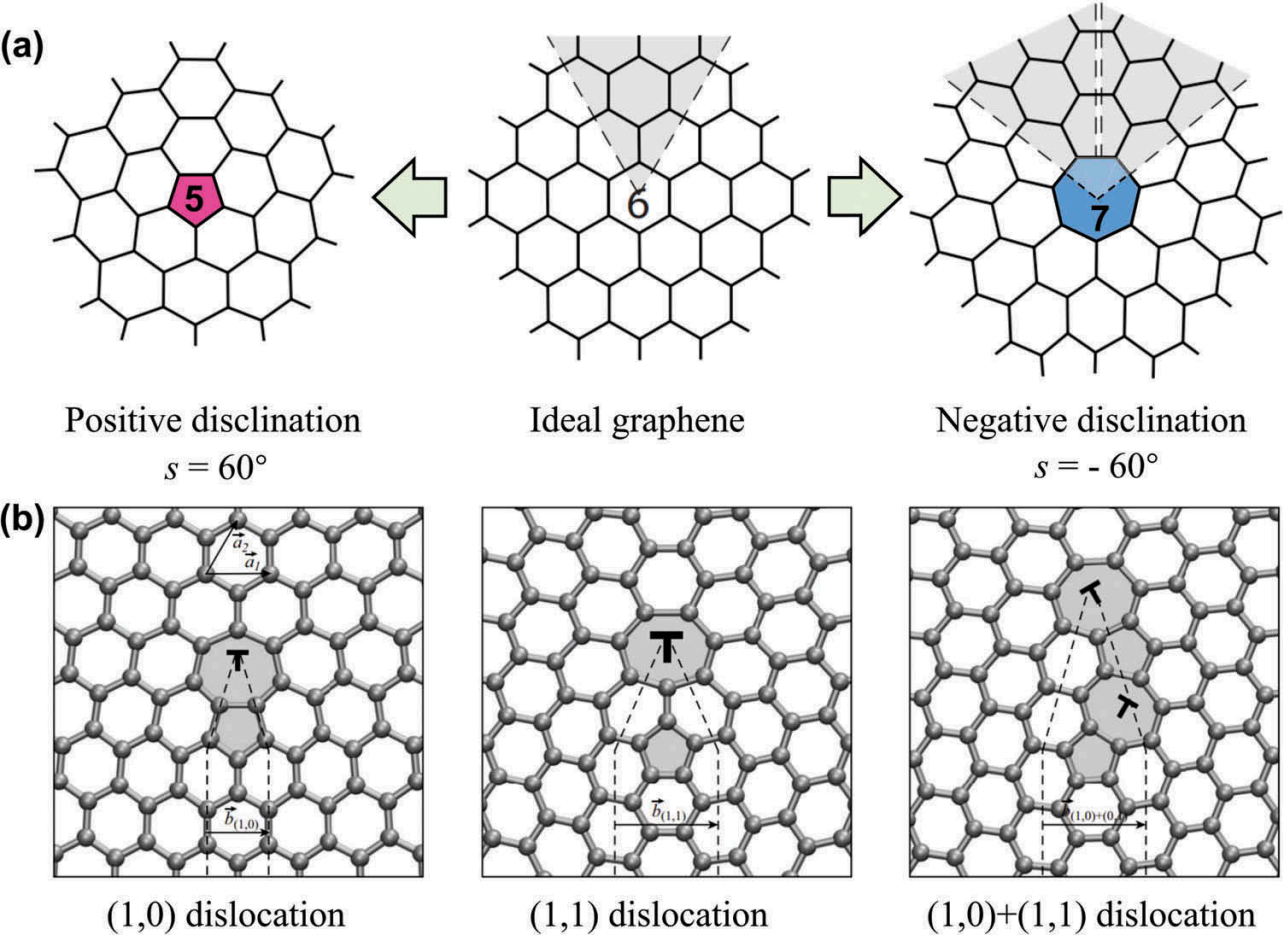
Configurations of (a) disclinations and (b) dislocations in graphene lattice [207] (reused with permission from [207] Copyright ©2010 American Physical Society.).

The graphenes produced via the CVD method are usually polycrystalline, due to the presence of topological defects: disclinations, dislocations and GBs, which are able to alter the lattice orientations. An intriguing feature of these topological defects is that they can exist in graphene without introducing local disorder into crystalline lattice. Figure 12(a) shows the configuration of disclinations which are elementary topological defects in the graphene sheet, resulting from the addition or removal of semi-infinite wedges. For example, the positive wedge angle (s = 60°) allows a pentagon to be embedded into the honeycomb lattice of graphene, while the negative wedge angle (s = −60°) creates a heptagon embedded in the graphene lattice. The isolated non-hexagonal rings in graphene inevitably result in non-planar structures.

As seen in Figure 12(b), dislocations are topological defects that are equivalent to the pairs of complementary disclinations. The topological invariant of a dislocation is the Burgers vector $\vec{b}$, which is a proper translational vector of graphene lattice:

(35) $$\vec{b}=m{\vec{a}}_{1}+n{\vec{a}}_{2}$$

where ${\vec{a}}_{1}$ and ${\vec{a}}_{2}$ are lattice vectors, and the pair of integers (m, n) is used as a description of the dislocation. The Burgers vector is related to the distance between two disclinations on graphene lattice. Dislocation (1,0) shows the smallest Burgers vector ($\left|\vec{b}\right|=2.46$ Å) that is achieved by an edge-sharing heptagon-pentagon dislocation which inserts a semi-infinite strip of atoms along the armchair high-symmetry direction in graphene. Whereas the (1,1) dislocation has a longer Burgers vector ($\left|\vec{b}\right|=4.23\;$ *Å*), and the semi-infinite strip is inserted along the zigzag direction. The core of dislocation with a Burgers vector the same as that of dislocation (1,1) can be also constructed from two dislocations, such as (1,0)+(1,1) dislocation pair.

**Figure 13.**
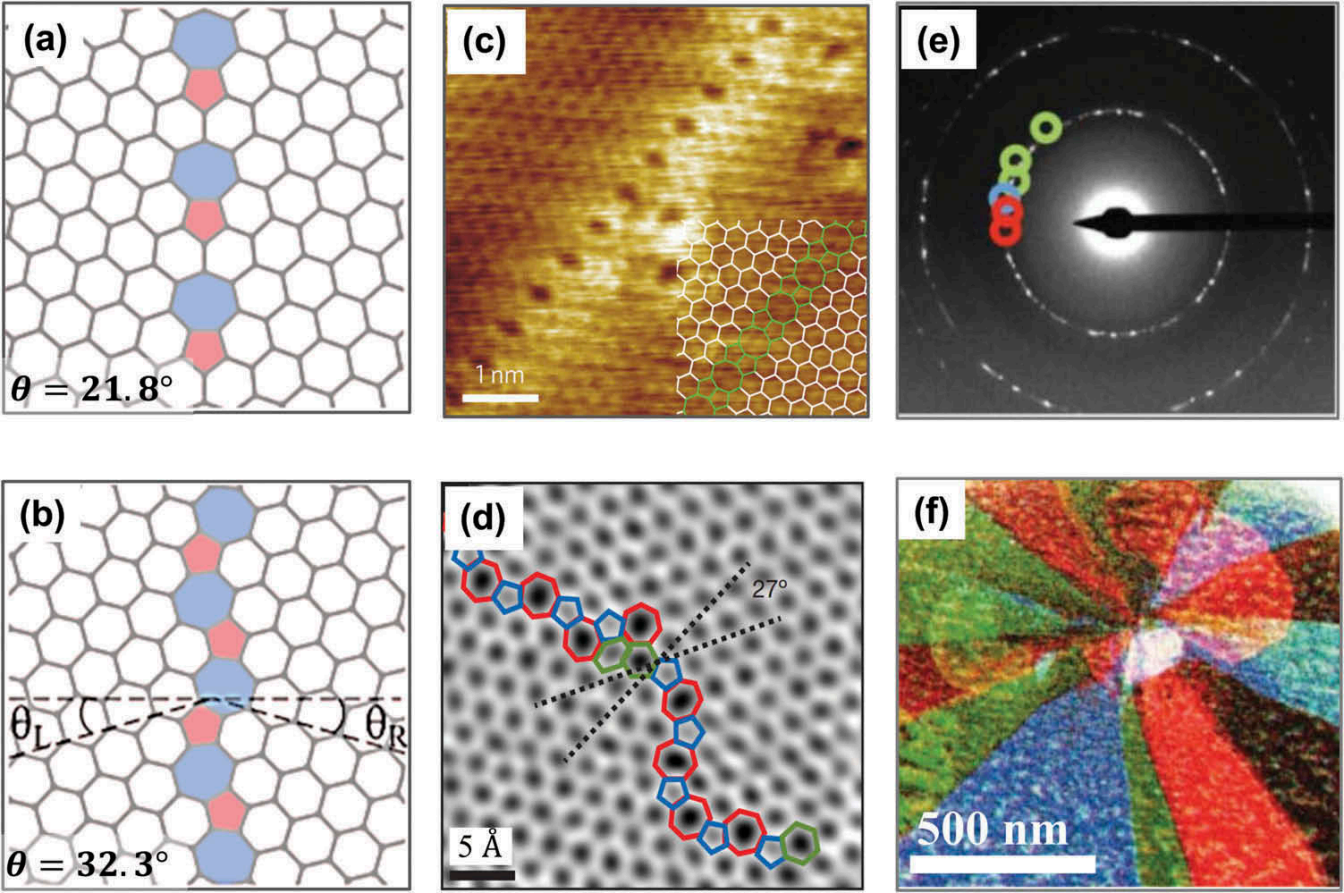
Configurations of (a) the *θ* = 21.8° and (b) the *θ* = 32.2° symmetric large-angle GBs, respectively [137]. (c) STM image of a regular line defect in graphene on the Ni(111) [208]. (d) Aberration-corrected annular ADF-STEM image of two grains which intersect with a relative rotation of 27°, and are stitched together by an aperiodic line of dislocations [209]. (e) Electron diffraction pattern obtained by DF-TEM imaging graphene grains one by one with few-nanometer resolution using an objective aperture filter in the back focal plane through a small range of angles, and repeating this process using several different aperture filters. (f) False-color, DF image overlay of the sizes, shapes and orientations of several grains [209] (reused with permissions from [137] Copyright © 2014, Springer Nature, [208] Copyright © 2010, Springer Nature, and [209] Copyright © 2011, Springer Nature.).

GBs can be viewed as a type of line defects formed by one-dimensional chains of aligned dislocations [205], which form the interface between the domains of material with different crystallographic orientations. The misorientation angle θ = θ_L_ + θ_R_ (0° < θ < 60°), describing the mutual orientation of the two crystalline domains, is a critical topological invariant of GBs. Figure 13(a,b) illustrate the configurations of the $\theta =21.8^{\circ }$ and the $\theta =32.2^{\circ }$ symmetric large-angle GBs, respectively. Small-angle GBs with misorientation angles close to 0° or 60° are thought to be along armchair or zigzag directions respectively. According to Frank’s equation [206], the misorientation angle resulted from aligning (1,0) dislocations along the grain-boundary line is related to the separation between the neighboring dislocations d and their Burgers vectors $\vec{b}$:

(36) $$\theta =2\arcsin\frac{\left|\vec{b}\right|}{2d}$$

Higher densities of dislocations correspond to smaller separations between neighboring dislocations, and consequently are expected to lead to larger misorientation angles. Further, the small-angle GBs in suspended graphene tend to form the out-of-plane buckling for further reducing their formation energies, while large-angle GBs are almost flat [207].

Both STM and SEM are suitable for the identification of GBs in graphene lattice. It is evidenced from the atomically resolved STM image (see Figure 13(c)) of a regular line defect in graphene on the Ni(111) that only three atoms rather than all six atoms are visible in the hexagonal lattice, which is attributed to the two diverse adsorption sites of carbon atoms on the Ni substrate [208]. It is noted that this line defect consists of one octagon and a pair of pentagons. Figure 13(d) shows the aberration-corrected annular dark-field STEM (ADF-STEM) image of two grains which intersect with a relative rotation of $27^{\circ }$, and are stitched together by an aperiodic line of dislocations [209]. As seen in Figure 13(f), the graphene grain structures can be visualized by repeatedly capturing DF-STEM images of grains using several different aperture locations (color-coded circles in Figure 13(e)), then coloring and overlaying these dark-field (DF) images [209]. The produced false-color, DF images overlay well depict the sizes, shapes and orientations of several grains in an area of interest. Furthermore, STEM can provide histograms of gain sizes and relative grain rotation angles for better understanding of the GBs [209,210]. It is also possible to visualize GBs by optical birefringence of graphene surface covered with nematic liquid crystal [211], by spectroscopic Raman imaging of the defect-activated D mode [78,212], and by infrared nanoimaging technique [213].

### 3.3. Vacancies, adatoms and sp^3^-defects

**Figure 14.**
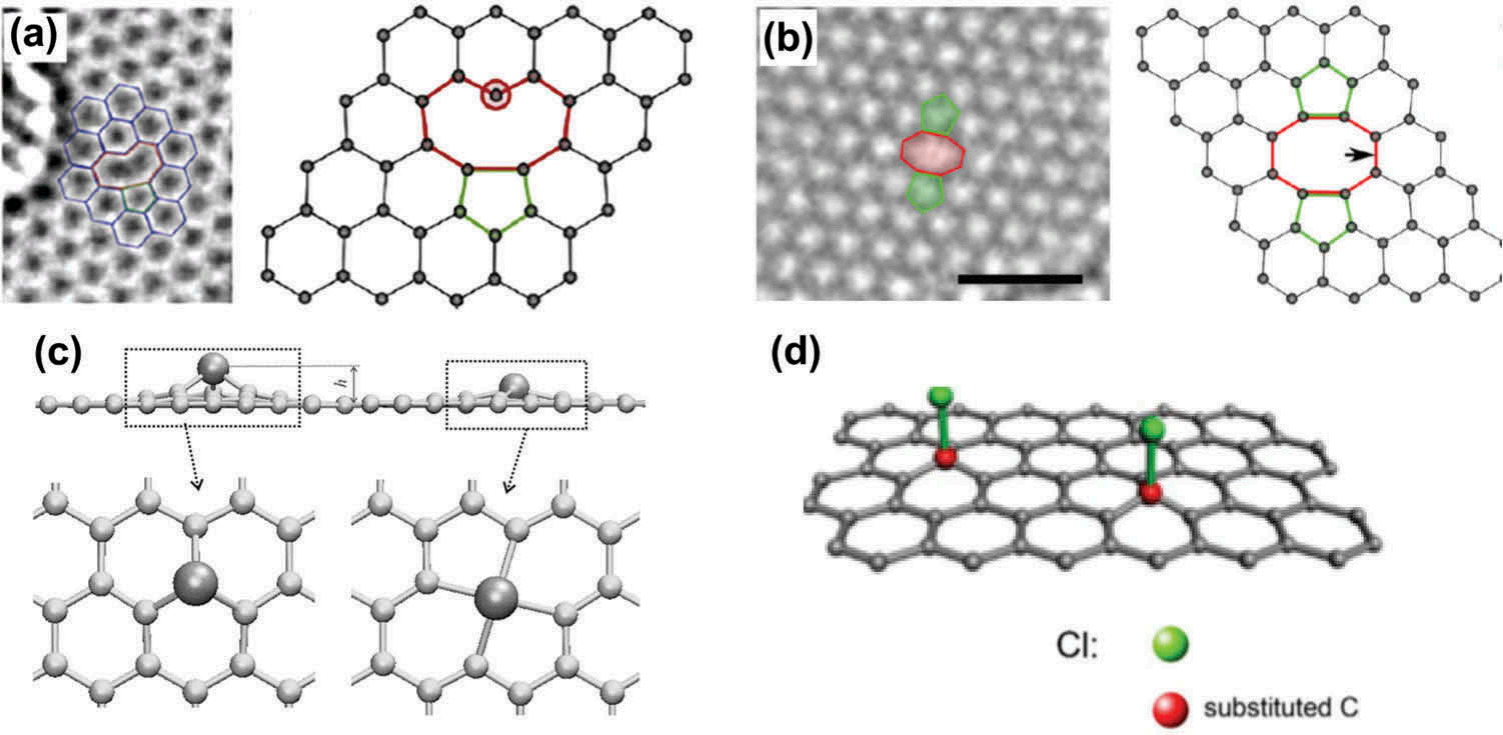
Configurations of (a) SV (5–9) [214], (b) double vacancy [214], (c) transition metal atoms adsorbed on single and double vacancies in a graphene sheet [216] and (d) sp^3^ defects [225] (reused with permissions from [214] Copyright © 2008, American Chemical Society, [50] Copyright © 2011, American Chemical Society, [216] Copyright ©2009 American Physical Society, and [225] © 2017 Elsevier Inc. All rights reserved.).

Other three types of common defects are vacancies, adatoms and sp^3^-defects, and their configurations can be seen in Figure 14. A single vacancy (SV) in the lattice refers to the single missing atom (see Figure 14(a)), which can be observed by TEM [214]. Due to the Jahn-Teller distortion, two of three dangling bonds are saturated and pointed towards the missing atom. One of them always remains because of geometrical reasons. The coalescence of two SVs will form a double vacancy, as seen in Figure 14(b). For a fully reconstructed double vacancy, two pentagons and one octagon appear, leading to no dangling bond. As a result, minor perturbations exist in the bond lengths around the defect. The simulation results also indicate that double vacancies are thermodynamically favorable as compared with SVs [215]. In fact, since vacancies with an even number of missing atoms allow the complete saturation of dangling bonds, they are energetically favored over defects with an odd number of missing atoms.

Transition-metal (TM) atoms adsorbed on perfect graphene sheets exhibit low migration barriers, in the range of 0.2–0.8 eV, indicating the high-mobility of adatoms at room temperature [216]. Hence, it is hard to achieve a controlled chemisorption of TM atoms on pristine graphene for manufacturing graphene-based Kondo systems. Figure 14(c) (left) illustrates the configuration of the adsorption of TM atoms on SVs in graphene sheet, where the adatoms are displaced outwards from the graphene plane with an elevation h of up to 2 Å, as the radii of TM atoms are larger than that of carbon atoms. For the adsorption of TM atoms on multiple vacancies in graphene sheet, based on the general crystal field theory, the interaction of impurity atoms with the ligand bonds is expected to be weaker when placed on a larger ‘hole’ in the graphene sheet, thus resulting in higher spin states of the complex.

The sp^3^ defects can be observed in hydrogenated [217], fluorinated [218,219], chlorinated [220,221] and oxidized graphenes [222]. Figure 14(d) shows the sp^3^ defects produced by the chlorination of graphene following the method described in a previous study [223]. Cl atoms were connected to the graphene sheets via covalent bonds (Cl–C) after a five-minute reaction. As the out-of-plane bonding with the atom introduces distortions in the crystal lattice, it is expected to induce both on-site and hopping defects. Furthermore, sp^3^ defects usually appear in form of dimers or clusters rather than being isolated [224].

### 3.4. Formation and migration energy of different structural defects

**Figure 15.**
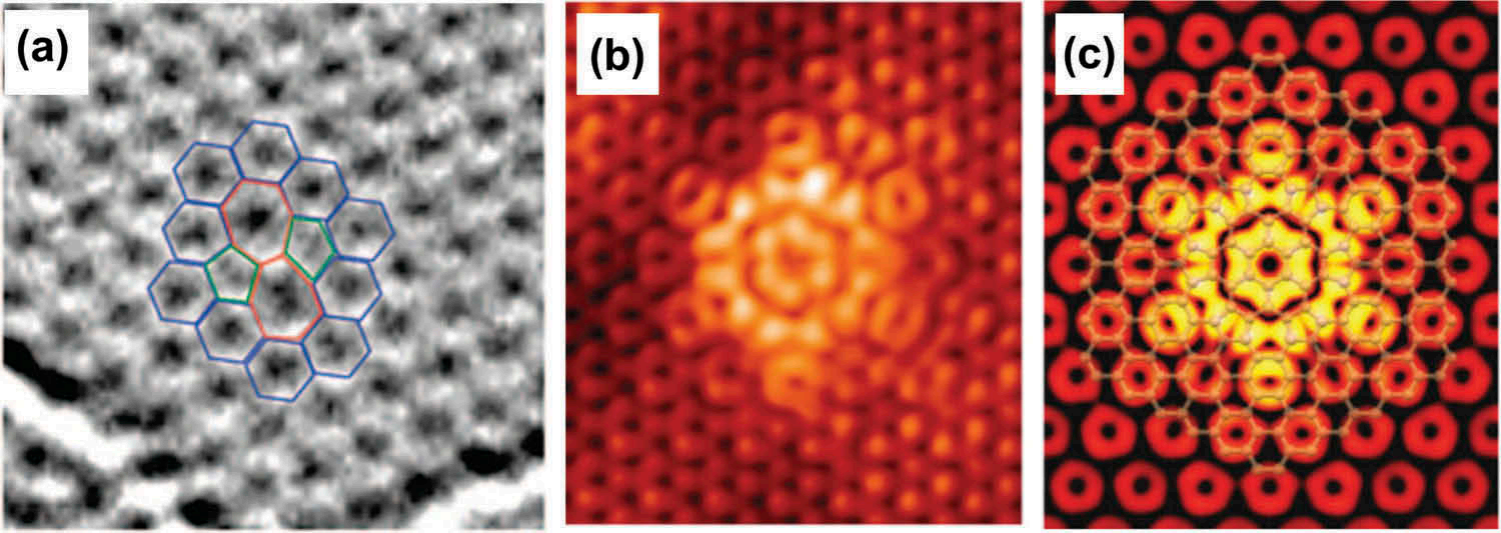
(a) TEM image of a SW defect, formed by rotating a carbon–carbon bond by 90° [214]. (b) STM topography of a sixfold defect observed in the growth of epitaxial graphene on SiC at −300 mV sample bias [228]. (c) Simulated STM image of the C6(1,1) defect using DFT calculations [228] (reused with permissions from [214] Copyright © 2008, American Chemical Society, and [228] Copyright © 2011 American Physical Society.).

Table 1 lists the formation energy of different types of defects in graphene sheets. The lower the formation energy the defect requires, the easier for it is to be generated in the lattice. For instance, the Stone–Wales (SW) defect exhibits a remarkably low formation energy of ~ 4.9 eV, so it is commonly seen in graphenes. Figure 15(a) indicates the TEM image of SW defects, which are created by transforming four hexagons into two pentagons and two heptagons by the rotation of a carbon–carbon bond by 90° [214]. Moreover, since SV configuration shows higher energy per missing atom as compared with the configuration of double vacancy, SVs are relatively unstable, and consequently less frequently observed in the graphene crystals. Apart from (5–8–5) configuration, double vacancies have another usual configuration (555–777) for accommodating two missing atoms, and the total formation energy of the latter is roughly 1 eV lower than that of the former. The migrations of adatoms and SVs result in the recombination of adatom–SV pairs. However, the resultant metastable configuration of adatom–SV pair is unstable due to its considerably high formation energy of 14 eV.

**Table 1.** Properties of various types of defects in graphene.

Defect Type	Configuration	N_core_	Formation Energy (eV)	Energy Per Dislocation(5–7 pair) (eV)	Migration Energy (eV)	Refs.
SW	55–77	2	4.9	2.5	10	[230–232]
Inverse SW	57–57	2	5.8	2.9		[231]
SV	5–9		7.3– 7.5		1.2– 1.4	[229]
Double vacancy	5–8-5		7.2– 7.9		7	[229,233]
	555–777		6.4– 7.5		6	[234,284]
Adatom			6– 7		0.4	[235]
Adatom–SV pair			14			[231]
Dislocation	$b=\left(1,0\right)$		7.5			[207]
Linear GB	$\theta =21.8^{\circ }$		0.338 eV/*Å*	2.2		[207,228]
	$\theta =32.2^{\circ }$		0.284 eV/*Å*	1.3		[207,228]
Rotational GB	C3	13	9.3	3.1		[228]
	C6(1,1)	24	7.0	1.2		[228]
	C6(2,1)	54	19.9	3.3		[228]

Isolated disclinations require out-of-plane deformations of the graphene sheet and therefore have high formation energies [137]. This makes them highly unlikely to be developed in monolayer graphene. The formation energy of the (1,0) dislocation is predicted to be about 7.5 eV and 6.2 eV by first-principles [207] and empirical force-filed calculations [226]. For small-angle ($\theta <10^{\circ }$) linear GBs constructed by a series of (1,0) dislocations, the grain-boundary energy can be well described by

(37) $$\gamma \left(\theta^{\prime }\right)=\frac{E_{f}\theta^{\prime }}{\left|\vec{b}\right|}$$

where $E_{f}$ is the formation energy of the (1,0) dislocation, $\left|\vec{b}\right|=2.46$ *Å* the length of Burgers vector for (1,0) dislocation, and misorientation angle $\theta =\theta^{\prime }$ or $\theta =60-\theta^{\prime }$ for armchair and zigzag small-angle GBs, respectively. In this case, the grain-boundary energy scales linearly with both $\theta^{\prime }$ and the formation energy of (1,0) dislocations [227].

The θ = 21.8° and the θ = 32.2° symmetric large-angle linear GBs have particularly low formation energies of 0.338 and 0.284 eV/Å, respectively (see Table 1). Among the rotational GBs, flower defect with the C6(1,1) configuration, as shown in Figure 15(b,c), exhibits the lowest energy per dislocation of 1.2 eV. In fact, it is lower than the energy of any other known topological defects in graphene. Such low-energy GB loops are generally created by the coalescence of mobile dislocations or SW defects.

It is noted that sometimes the defects in the crystal structure of graphene are migratory rather than stationary, and their migration may influence the properties of a defective crystal, especially the annealing and reconstruction behaviors. Each defect shows a certain mobility parallel to the graphene plane. For instance, the mobility for SW defects and divacancies are immeasurably low, while the mobility for adatoms is fairly high. The migration of each defect is governed by an activation barrier which is indicated by the migration energy. Therefore, the migration is expected to increase exponentially as temperature increases. For a SV in graphene, the calculated migration energy ranges from 1.2 to 1.4 eV [229], which allows a measurable migration at a relatively low temperature (100–200 ℃).

### 3.5. Effects of disorders on properties of graphene

#### 3.5.1. Electrical properties

In an idealistic planar graphene model without disorders, the Fermi energy level lies at the Dirac point, where the valence band and conduction band intersect, and the dispersion relation around the Dirac point is isotropic and linear [236]. However, the electronic homogeneity of graphene would be violated by the introduction of disorders into the graphene structure. These disorders are able to alter the bond length of the interatomic bonds and lead to the re-hybridization of σ and π orbitals. Moreover, all defects may cause the scattering of electron waves and change the electron trajectories [237,238]. As a result, the electronic structure in the vicinity of these disorders differs from that in a perfect lattice. More specifically, intrinsic ripples are expected to influence the electrical properties of graphene by changing band gap [239], creating polarized carrier puddles [240] and inducing pseudo-magnetic fields [241]. Whereas wrinkles and crumples result in several electronic phenomena, such as electron-hole puddles [189,242], carrier scattering [195,243], band gap opening [244], suppression of weak localization [245] and quantum corrections [246].

Disclinations and dislocations induce distortions in the graphene lattice which probably alter the carbon–carbon bond length, and consequently the band structure is changed. Due to the strong scattering of charge carriers, GBs can impede electronic transport, thus degrading the electrical properties (e.g. decreasing mobility and increasing resistance) of polycrystalline graphene [78,247–250]. In addition, GBs with larger grain size exhibit relatively better conductive performance [247,251]. On the other hand, a few experiments [209,252,253] observed that the configuration of GBs or the variation of grain sizes had little effects on the conductive properties of graphene. Recent study [254] also suggested that increasing grain size would be an inefficient way to improve the electrical conductivity of graphene when the grain size is larger than 1 μm. Intriguingly, because of a large number conducting channels along the grain-boundary line, the conductivity in this direction may be enhanced [208].

Point defects such as SW defects, vacancies, and adatoms can serve as scattering centers for electron waves, and thus reducing the conductivity of graphene [255–258]. The charged impurities adsorbed on graphene or located at the interface between graphene and substrate induce Coulomb scattering [259], and are responsible for the electron-hole puddles at the neutrality point [260,261]. Both the resonant scattering [262] and Coulomb scattering significantly influence the drift mobility and electron mean free path, and therefore change the electrical properties of graphene [263]. Doping by substitutional impurities (e.g. nitrogen atoms and boron atoms) is a straightforward way to broaden the van-Hove singularities in the DOS and to shift the Fermi level [264], and these doped graphenes can be characterized by Raman spectroscopy [265]. Chemical bonding of impurities like hydrogen or fluorine on graphene sheet may generate a local distortion of the hexagonal lattice and lead to spin–orbit coupling [266].

#### 3.5.2. Thermal properties

The room-temperature in-plane thermal conductivity of suspended monolayer graphene is among the highest of any known materials, 4840–5300 W/(m K), as determined by micro-Raman thermometry [15]. However, the thermal conductivity of graphene significantly decreases when it is in contact with a substrate such as SiO_2_ [267] or confined in GNRs [268], due to the high-sensitivity of the phonon propagation in an atomically thin graphene sheet to surface or edge perturbations [269]. Numerical simulations [270] indicate that the scattering of phonons by defects and delocalized interaction between them lead to a transition of thermal transfer process from propagating mode to diffusive modes. Consequently, the thermal conductivity of graphene strongly depends on the concentration of SVs and SW defects. Haskins et al. [271] also observed substantial reduction in thermal conductivity of graphene due to the introduction of a variety of randomly oriented and distributed defects, such as SVs, divacancies and SW defects. Typically, SVs caused the largest reduction of lattice thermal conductivity due to their less stable two-coordinated atoms [271]. Besides, zigzag GNRs are found to be more thermally conductive than armchair GNRs, given that their width and length are the same [272–274]. A high surface roughness at the edges of graphene notably shortens the phonon mean free path, and thus deteriorates the thermal conductivity. At room temperature, approximately 80% reduction was observed in the thermal conductivity of GNRs with edge roughness value of 7.28 Å, as compared to that of smooth-edge ribbons of the same size [271].

Due to the scattering effect induced by GBs, the thermal conductivity of CVD-grown polycrystalline graphene is generally lower than that of exfoliated graphene [275]. In non-equilibrium MD simulations, Bagri et al. [276] found a jump in temperature at tilt GBs when a constant heat flux was applied, and they calculated the boundary conductance by relating the jump in temperature to the heat flux. The boundary conductance decreased with increasing misorientation angles of GBs, as large misorientation angles corresponded to higher density of dislocations. It is also noted that the thermal conductivity of the polycrystalline graphene was dominated by the scattering of phonons within the grains when grains are very large in size, but primarily determined by scattering from GBs when grain size decreased to a considerably low-value. Especially, when the grain sizes are smaller than phonon mean free path (about a few hundred nanometers), the type and size of GBs are expected to significantly influence the boundary conductance [277]. In this case, single GB yields transmission from 50% to 80% of the ballistic thermal conductance. Further, GBs consisting of octagon rings have lower thermal transmission than that of regular GBs with pentagon and heptagon pairs [277]. In practice, defects such as vacancies and voids tend to segregate at GBs [209,210], which are expected to further lower the thermal conductance of the boundaries.

#### 3.5.3. Chemical properties

Defect-free graphene surfaces appear to be chemically inert, and these surfaces usually interact with other molecules via physical adsorption (π−π interactions). However, the graphene edges that contain hydrogen seem to be more reactive, and thus several chemical groups (e.g. hydroxyl, carboxyl, hydrogenated and amines) can be anchored at these edges. In addition, the reactivity of graphene edges is nontrivial and sensitive to the carbon terminations (either armchair or zigzag), due to the delicate competition of energy per atom and their density [278]. In corrugated graphene with high degrees of curvature, the chemical reactivity of graphene surface is notably enhanced, especially when the ratio of height to radius for corrugations (e.g., ripples and wrinkles) is higher than 0.07 [279]. Besides, the highly crumpled graphene exhibits super-hydrophobicity and tunable wettability [201]. The chemical reactivity of graphene can be enhanced by introducing structural defects associated with dangling bonds. Indeed, it is suggested by the numerical simulations [280,281] that hydroxyl, carboxyl and other groups can be easily attached to vacancy-type defects. Reconstructed defects without dangling bonds, such as SW defects and divacancies, have the possibility of increasing local reactivity [282] due to the locally changed density of π-electrons [280,283]. It is evidenced from experiments that metal atoms may be trapped in the reconstructed vacancies [284]. Substitutional non-carbon atoms embedded in the graphitic lattice, such as nitrogen and boron dopants, possess more or less valence electrons than that of carbon atoms, and thus increase the surface reactivity [285]. Moreover, nitrogen-doped graphene is an efficient electrocatalyst for reduction processes [286,287]. Another efficient way for making graphene sheet less inert is by reacting it with halogen atoms such as chlorine [220,221] and fluorine [218,219]. Finally, attaching oxygenated groups on the sp^2^ hybridized surfaces enables the resulting graphene materials to be hydrophilic and more reactive [288].

#### 3.5.4. Mechanical properties

Due to the extremely strong in-plane σ bonds between neighboring carbon atoms in the honey-comb lattice, defect-free monolayer graphene exhibits ultra-high elastic modulus and unsurpassed tensile strength [7,14,289], which can be measured by AFM-based nanoindentation. However, defects seem to be ubiquitous in practical graphene devices, and the mechanical properties of graphene are supposed to be affected by these defects in different ways, depending on the density and type of defects. Typically, the out-of-plane deformations, like wrinkles, enhance the adhesion between graphene and the underlying substrates [290]. Atomistic finite element analysis (FEA) results [291] reveal that though one SV insignificantly reduced the effective elastic modulus of graphene sheet, increasing the number of SVs can cause a strong reduction. Besides, the shear modulus and Poisson’s ration of defective graphene sheet are closely related to the position of SVs, and a pronounced reduction is observed when SVs are in a region of large strain gradient. Another atomistic FEA study [292] indicates that increasing the density of SW defects in graphene can change the Young’s modulus dramatically, particularly when the distance between neighboring defects is smaller than 2 nm (diameter of interaction region of SW defects [293]). Besides, the presence of single SW defects can result in significant reduction of the ultimate strength for graphene sheet, and further strength reductions are predicted for cases where adjacent SW defects are interacting with each other. In this case, the reduction of ultimate strength is governed more by the separation distance rather than the defect density. More theoretical studies [294–296] were carried out for investigation the effects of point defects (e.g. SVs, divacancies and SW defects) on the mechanical properties of graphene sheet.

In addition, MD simulations [297] suggest that compared with Young’s modulus, the tensile strength and fracture strain of hydrogenated graphene have higher sensitivity to the functionalization. The dramatic deterioration in mechanical properties is attributed to not only the conversion of sp^2^ to sp^3^ bonding but also the easy-rotation of unsupported sp^3^ bonds. On the other hand, it is reported [298] that the 2D elastic modulus and strength of graphene can be maintained even at a high-density of sp^3^-type defects, in contrast to significant degradation of mechanical properties in the vacancy-defect regime.

In polycrystalline graphene, the Young’s modulus and fracture strength are more sensitive to the variations of temperature and strain rate than that in monocrystalline graphene [299,300]. Besides, the decrease of gran size (from 10 nm to 2.5 nm) is expected to lead to the drop of Young’s modulus and fracture strength. As large-angle GBs are able to better accommodate the stained seven-membered rings in graphene lattice, they have higher strengths than the low-angle counterparts, and even as strong as the pristine graphene structure [93]. More studies regarding the effects of grain boundaries on the mechanical properties of graphene can be found in Refs [301–304]..

### 3.6. Generation of disorders in graphene preparation procedures

**Figure 16.**
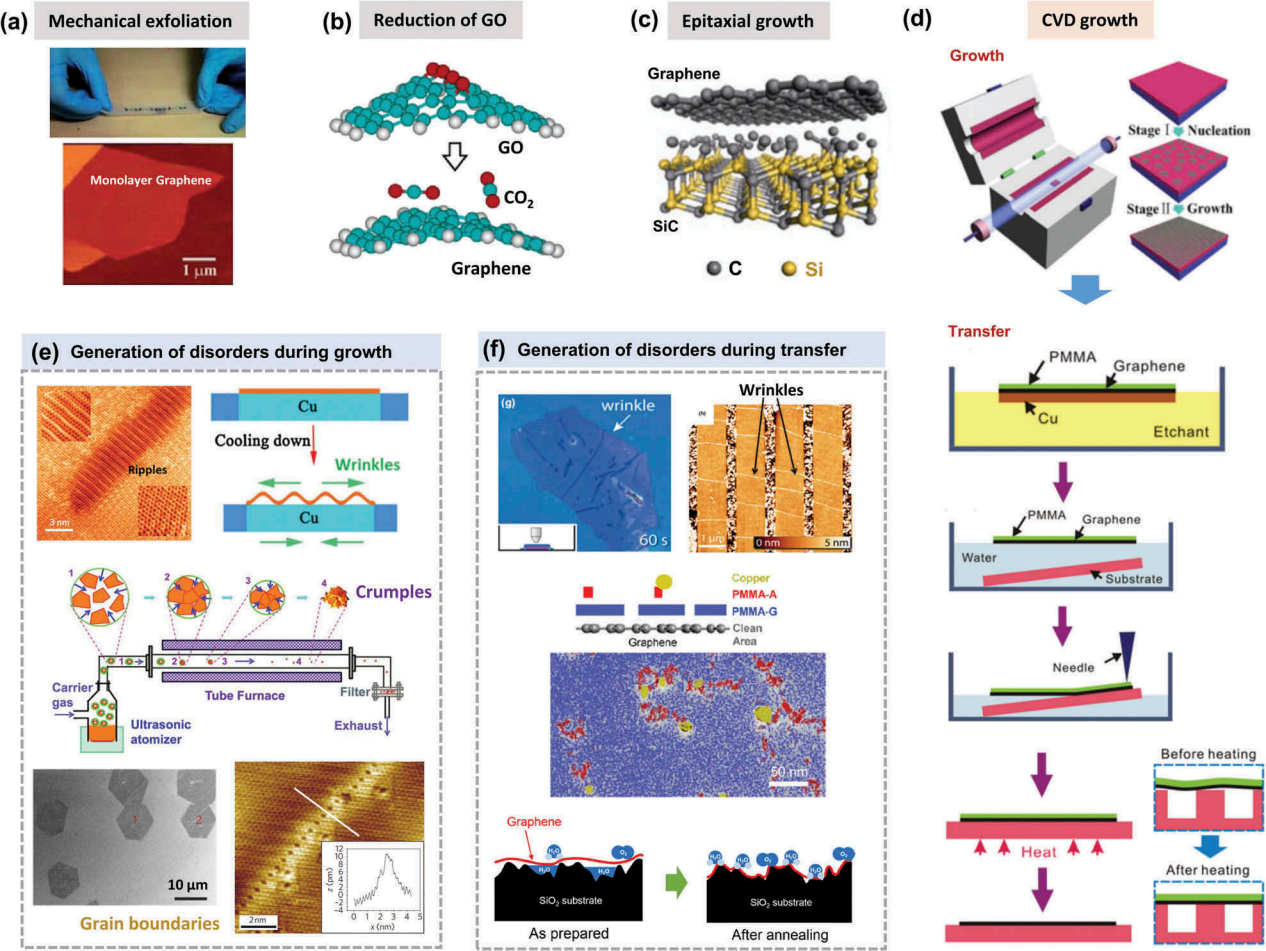
Fabrication of monolayer graphene by (a) mechanical exfoliation [320], (b) reduction of GO [353], (c) epitaxial growth [354] and (d) CVD growth [321,312]. Generation of disorders during (e) growth [240,192,78,208] and (f) transfer [319,329,322]. In (f), PMMA-A and PMMA-G correspond to PMMA facing the air and graphene respectively. (reused with permissions from [320] Copyright © 2015, Royal Society of Chemistry, [353] Copyright © 2006, American Chemical Society, [354] Copyright © 2013, Tsinghua University Press and Springer-Verlag Berlin Heidelberg, [321] Copyright © 2015, Elsevier. [312] Copyright © 2011, American Chemical Society, [240] Copyright © 2012, Springer Nature, [192] © 2015 The Authors. Published by Elsevier Ltd, [78] Copyright © 2011, Springer Nature, [208] Copyright © 2010, Springer Nature, [319] Rights managed by AIP Publishing, [329] Copyright © 2012, American Chemical Society, and [322] © 2017 Elsevier Ltd. All rights reserved.)

Disorders can be deliberately introduced into the graphene lattice for tailoring properties by additional treatment, which will be discussed in the next part. Besides, it is possible to bring defects into the crystal structure of pristine graphene during preparation process. Up to now, a large number of methods have been developed for fabrication of graphene (see Figure 16(a-d)). These methods can be categorized into two major classes, namely top–down methods (e.g. graphite exfoliation [6] and reduction of GO [305]) and bottom–up methods (e.g. epitaxial growth [306] and CVD [17,21]). Although mechanical exfoliation of HPOG by using Scotch tape allows the preparation of ultra-clean free-standing monolayer graphene, this method is extremely labor intensive and remains unfeasible for the production of large-area graphene sheets. By contrast, self-assembly of reduced graphene oxide (rGO) demonstrates the possibility of low-cost and large-scale synthesis of transparent films. However, a large number of defects, including point defects, line defects and adsorption of functional groups, which are formed during the oxidation, vigorous exfoliation and reduction processes are introduced into these assembled graphene films. Epitaxial growth on silicon carbide [306–308] or ruthenium [309] at high-temperatures in ultrahigh vacuum can provide high-quality graphene with a size as large as that of the substrate [310]. However, the produced graphene strongly interacts with the substrate, hindering fabrication of electrically isolated monolayer graphene. On the other hand, CVD growth of graphene on catalytic metals, such as Cu [21] and Ni [17,21,311], is a promising approach for efficient large-scale production of defect-free graphene with controllable number of layers. Moreover, the resulting monolayer graphene can be easily transferred to arbitrary substrates [312]. Therefore, this section would concentrate on the generation of defects during graphene synthesis in the CVD process.

Fabrication of graphene by CVD comprises two steps: CVD-growing graphene on metal substrates and transferring graphene to a desired substrate. In the growing step, high-temperature growth facilitates the rapid annealing of defects, and thus generates less defects than low-temperature growth. Due to the high formation energy of vacancies and fast migration of adatoms, isolated vacancies are uncommonly seen in graphene sheets after growth. However, graphene spontaneously grows at different positions on the substrate surface, and consequently grain boundaries are formed at the amalgamation areas, leading to polycrystallinity. These GBs or other small topological defects generate in-plane stress, which is responsible for the out-of-plane deformations (ripples and wrinkles) [313]. Normally, the pattern of ripples is dependent on the temperature and the size of graphene sheet. As graphene is cooled at the end of growing step, the large difference between the thermal expansion coefficient (TEC) of graphene and substrates would result in isotropic and self-similar wrinkles [239,314]. Crumples are formed as a consequence of multidirectional forces applied on graphene during rapid evaporation, and they depend on the original size of the sheet and the compression force on the graphene sheet.

The graphene transfer is commonly executed by spin-coating a polymer layer (e.g. polymethylmethacrylate (PMMA)) to support the graphene while etching the metal catalyst away in an etchant [312]. Polymer-on-graphene layer is then transferred to a specified substrate, followed by dissolution of the polymer layer. However, the removal of polymer can be problematic, as it is technically inevitable to avoid the existence of physisorbed PMMA residues on the graphene surface. Apart from PMMA residue, the adsorbed O_2_ and trapped H_2_O also significantly influence the mobility of the transferred graphene [315]. The CVD-grown graphene is usually transferred from catalytic metals (e.g. Cu and Ni) to an insulating substrate such as SiO_2_ for practical applications (e.g. nanoelectronic devices). The graphene corrugations observed by a novel combined SEM/AFM/STM technique are suggested to be attributed to the partial conformation of the graphene to the SiO_2_, not to the intrinsic corrugations of graphene [316]. Besides, species like H_2_O may be trapped at the graphene/SiO_2_ interface [317,318]. Water drainage between graphene and substrate also plays a significant role in the formation of wrinkles [319].

Most of these introduced defects are undesirable, and somewhat exert negative impacts on the properties of graphene. Therefore, there is a need to provide approaches for removal of these disorders. Typically, the intrinsic ripples may be suppressed by depositing graphene on atomically smooth substrates [323]. Wrinkles can be reduced by increasing the thickness of nickel substrate at the growth step, soaking PMMA/graphene film in deionized water before transfer [194], and transferring graphene to hydrophobic substrates [319]. The crumpling degree of graphene film can be reversibly controlled by adhering graphene on a biaxially pre-stretched polymer substrate with controllable relaxation of the pre-strains in a particular order [201]. The line defects (or GBs) are expected to be avoid by controlling the nucleation of graphene grains using seeded growth, and to synthesize spatially ordered arrays of graphene grains with pre-determined locations [78]. Lastly, cleaning of transferred graphene surface can be efficiently done by annealing in a clean or reducing environment such as vacuum, H_2_/N_2_ or H_2_/Ar [316,324,325].

## 4. Modulation of structural defects in graphene

Disorders are predominantly brought in during the production process, and unavoidably occur due to the interaction with substrates and environment. As discussed in Section 3.5, the properties of graphene are sensitive to these intrinsic or extrinsic disorders. Sometimes these defects need to be removed for maintaining remarkable properties of pristine graphene. However, in most cases the properties of graphene are supposed to be tailored for satisfying different requirements in industries. Tuning properties of graphene can be realized by several defects modulations approaches, including particle irradiation [326–328], thermal annealing [329,330], chemical reaction [331,332] and strain treatment [333,334], as summarized in Table 2.

**Table 2.** Summary of several approaches for defects modulation in graphene crystals.

Approaches	Key Content	Substrate	Induced Disorders	Refs.
Particle Irradiation	Ar^+^ ions	Bilayer graphene	Interstitials and vacancies	[326]
	Ar^+^ ions	SiO_2_-supported monolayer graphene	Vacancies and substitutional Impurities	[336]
	α-beams (He^2+^)	Monolayer graphene	Vacancies, C = O and C–OO bonds	[327]
	Electrons	Monolayer CVD graphene	Vacancies, complex closed-loop defects, and dislocation pairs	[328]
	Electrons	Mechanically exfoliated monolayer graphene	Polygons and low-energy multivacancy	[339]
Thermal Annealing	~ 200 °C	CVD graphene	sp^3^ defects and partially formed radical sites	[329]
	500–1000 °C	rGO	Free radicals and oxygen groups	[330]
Chemical Reaction	CO and NO molecules	Monolayer graphene after irradiation	SV and N-doping	[331]
	Fluorinated maleimide molecules and a toluene solution	Monolayer and bilayer epitaxial graphene	sp^3^-defects and standing-wave patterns	[332]
	NH_3_ plasma	Polycrystalline graphene	Pyridine-like N, pyrrolelike N, and nitrites (NOx)	[347]
Strain Treatment	Uniaxial strain	Polycrystalline graphene	Lattice distortion, Grain boundaries	[333]
	Shear strain	CVD graphene	Wrinkles, transverse conducting channels and grain boundaries	[334]

### 4.1. Particle irradiation

Irradiation usually introduces disorders and leads to the self-organization or self-assembly in nanostructured carbon materials [335]. Typically, irradiating graphene with energetic particles, such as ions [326,336–338] or electrons [328,339], can effectively create point defects (mostly vacancies), due to the ballistic ejection of carbon atoms. Carbon atoms that gain sufficient energy (approximately 18–20 eV) from the irradiating beams may be sputtered away from graphene, get adsorbed on the sheet or migrate on the surface as adatoms.

**Figure 17.**
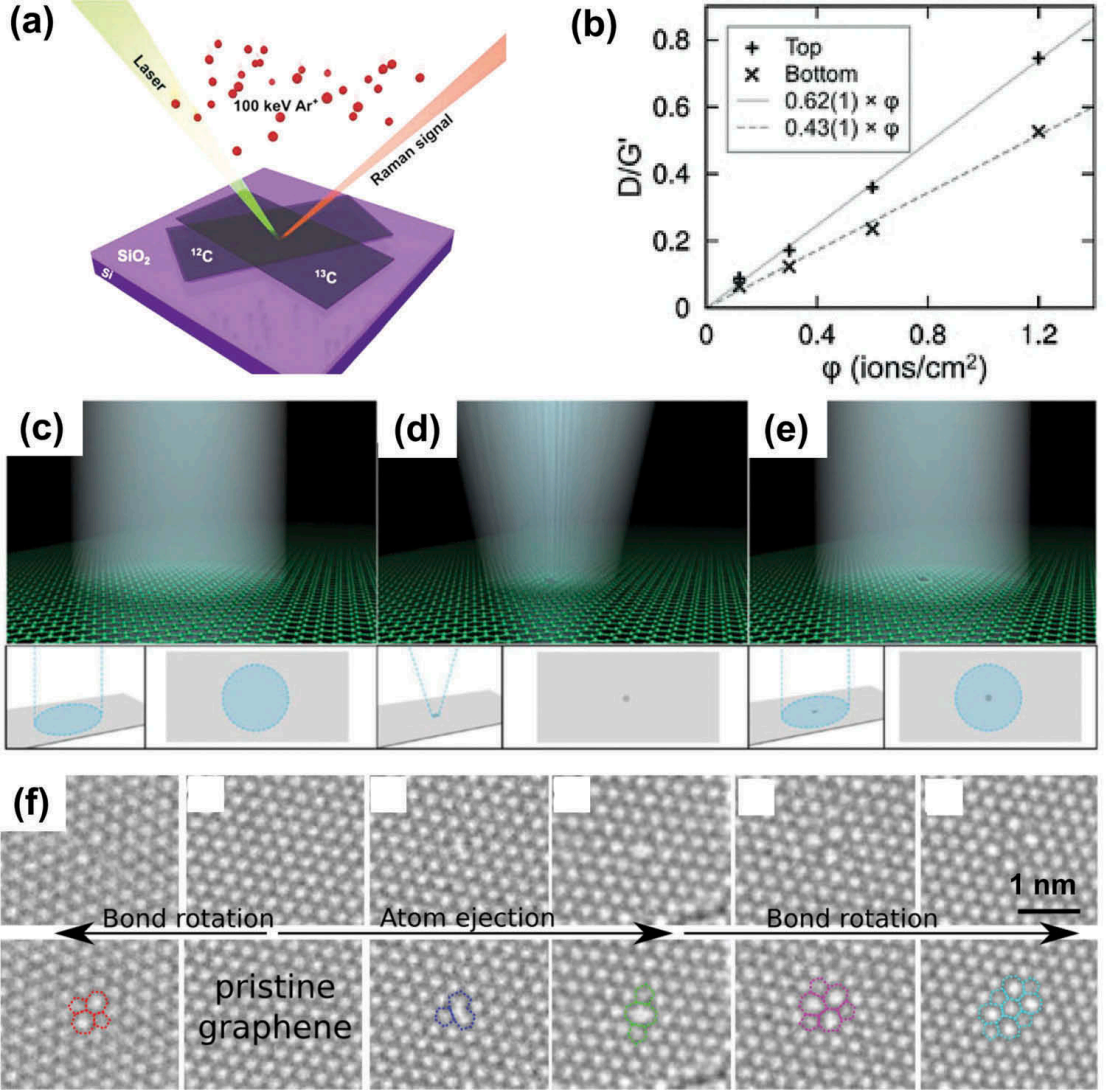
(a) Schematic illustration of the experimental setup for the irradiation of Ar+ ions with various doses followed by Raman probing [326]. (b) D/G’ intensity ratios as functions of Ar+ ion irradiation fluence (in 10^13^ ions/cm^2^) [326]. Schematics of the beam profile used in the three principle stages [328]: (c) a broad beam used to image graphene before defect formation, (d) a focused beam with a high current density used to form defects and (e) a broad beam used to image graphene after defect formation. (f) Creation of the defects can be explained by atom ejection and reorganization of bonds via bondrotation [339] (reused with permissions from [326] Copyright © 2012, John Wiley and Sons, [328] Copyright © 2012, Springer Nature, and [339] Copyright © 2011 American Physical Society).

Ion irradiation can be employed to selectively create defects as well as to pattern or mill graphene sheet by utilizing a focused ion beam (FIB) [340]. As seen in Figure 17(a), Kalbac et al. [326] created interstitials and vacancies on isotopically labeled bilayer graphene sheets by Ar+ ion irradiation with various doses. The amount of damage in the samples is assessed by Raman spectroscopy, and the experimental results indicate that the number of the vacancies shows a positive dependence on the intensity of ion dose, and the final defect density in the bottom layer was lower than that in the top layer (see Figure 17(b)). Interestingly, in α-beams (He^2+^) irradiation, a sufficient number of vacancies may result in the hole doping of graphene due to the charge interaction between broken carbon bonds and ambient O_2_ molecules, and the increase of work function due to hole doping showed a logarithmic behavior with respect to the irradiation dose [327]. Buchheim et al. [337] found that irradiation of He^+^ at energies (10–30 keV) allows the passage of more than 97% He^+^ particles without creating vacancies on the hexagonal lattice of freestanding graphene, which was corroborated by Raman spectroscopy. In contrast, large Ga^+^ ions at energies (5–30 keV) collide more often with the graphene lattice, and impart a notable higher sputter yield of ~ 50%.

However, defects created by ion beams are sporadically formed over a wide area of graphene, which lacks nanoscale spatial control and *in situ* monitoring. Alternatively, it is possible to *in situ* monitor the defects fabrication at atomic level by using an aberration-corrected TEM [328] following three principle steps (see Figure 17(c–e)): a broad electron beam used to image the pristine graphene with relatively low beam current density of ~ 10^5^ e^−1^ nm^−2^ s^−1^, a focused beam with high current density (10^8^ e^−1^ nm^−2^ s^−1^) employed to create defects, and a broad beam with low current density adopted to observe the formed disorders on graphene. It is evidenced from the electron irradiation experiments that both the location and average complexity of defect formation in graphene could be controlled by tailoring the electron beam current density and the exposure time. Some of the created defects (i.e. closed-loop defects) were stable, whereas others (i.e. SVs and divacancies) relaxed to simpler structures through bond rotations and surface adatom incorporation. Kotakoski et al. [339] investigated the bonding behavior of carbon and dynamics of defects in electron-irradiated graphene by observations under aberration-corrected HRTEM, along with DFT calculations. They found that electron beam can be used to selectively suppress or enhance bond rotations and atom removal in graphene, and the growth of SW defects and low-energy multivacancy structures can be explained by atom ejection and reorganization of bonds via bond rotation, as seen in Figure 17(f).

### 4.2. Thermal annealing

**Figure 18.**
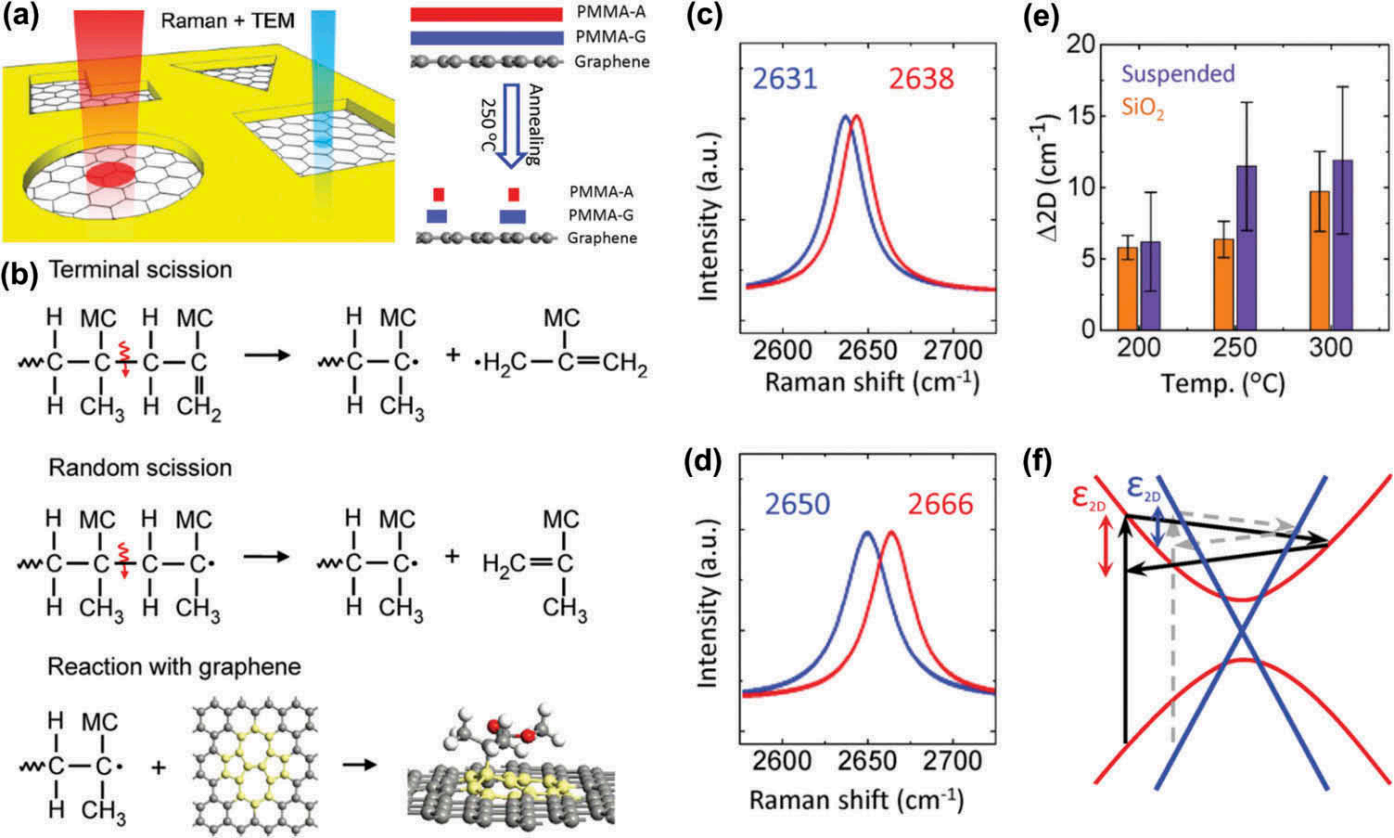
(a) Schematic illustration of a combination of TEM observation and Raman spectroscopy for graphene. PMMA-A and PMMA-G correspond to PMMA facing the air and graphene respectively. Comparisons of the 2D peak positions before and after annealing for (c) Si-supported and (d) free-standing CVD graphenes. (e) Histogram of the Δ2D as a function of annealing temperature for both Si-supported and free-standing graphenes. (f) Schematic illustration of the electronic structure near the Dirac points of pristine (linear) and annealed (parabolic) CVD graphenes. All pictures are extracted from Ref [329] (reused with permission from [329] Copyright © 2012, American Chemical Society.).

Thermal annealing of graphene in certain environment allows the removal of lattice defects and the restoration of graphitic structure. For e.g. surface contamination by polymer residues in graphene transfer step can be partially reduced by annealing at vacuum or reducing environment, as mentioned in Section 3.6. The sensitivity of electronic structure of graphene to the removal of polymer residue can be harnessed to tailor the properties of graphene. Generally, thermal degradation of polymers like PMMA is a complex radical chain reaction [341], which proceeds in three steps [342], as seen in Figure 18(b). Lin et al. [329] employed TEM in combination with Raman spectroscopy to study the thermal decomposition of PMMA (see Figure 18(a)). The decomposition temperature was lower for PMMA facing the air (PMMA–A) but higher for PMMA facing the graphene (PMMA–G). Experimental results reveal that the interaction between the thermally generated free carbons radicals on the graphene sheet and the polymer chains leads to the sp^3^-hybridization of carbons when annealing over a long period or at a high-temperature of 200 °C, due to the random scission of polymer chains. The rehybridization alters graphene’s band structure near the Fermi level (see Figure 18(f)), and the reduced Fermi velocity is responsible for the 2D blue-shift after annealing (see Figure 18(c,d)). Further, it is evidenced from Figure 18(e) that suspended graphene seems to be more sensitive to temperature than SiO_2_ supported graphene, and the 2D blue-shift is more significant at higher temperature. In the annealing treatment of rGO [330], a smaller amount of free radicals was created when rGO was annealed at low-temperature. As the annealing temperature was increased from 500 to 1000 °C, the amount of oxygen groups on the graphene surface decreased. As a result, the adjacent rGO layers got increasingly closer to each other, leading to the improvement of electrical conductivity between layers [343]. Conductivity measurement confirmed that the conductivity was lower when less free radicals were distributed in a two-dimensional ordered phase. Therefore, thermal annealing can reduce the oxidation level of rGO in a controlled manner for obtaining desired defects, conductivity, capacitance and surface reactivity. It is also found that thermal annealing in the presence of a hydrocarbon gas makes the high-conductive rGO accessible by defect healing [344].

### 4.3. Chemical reaction

**Figure 19.**
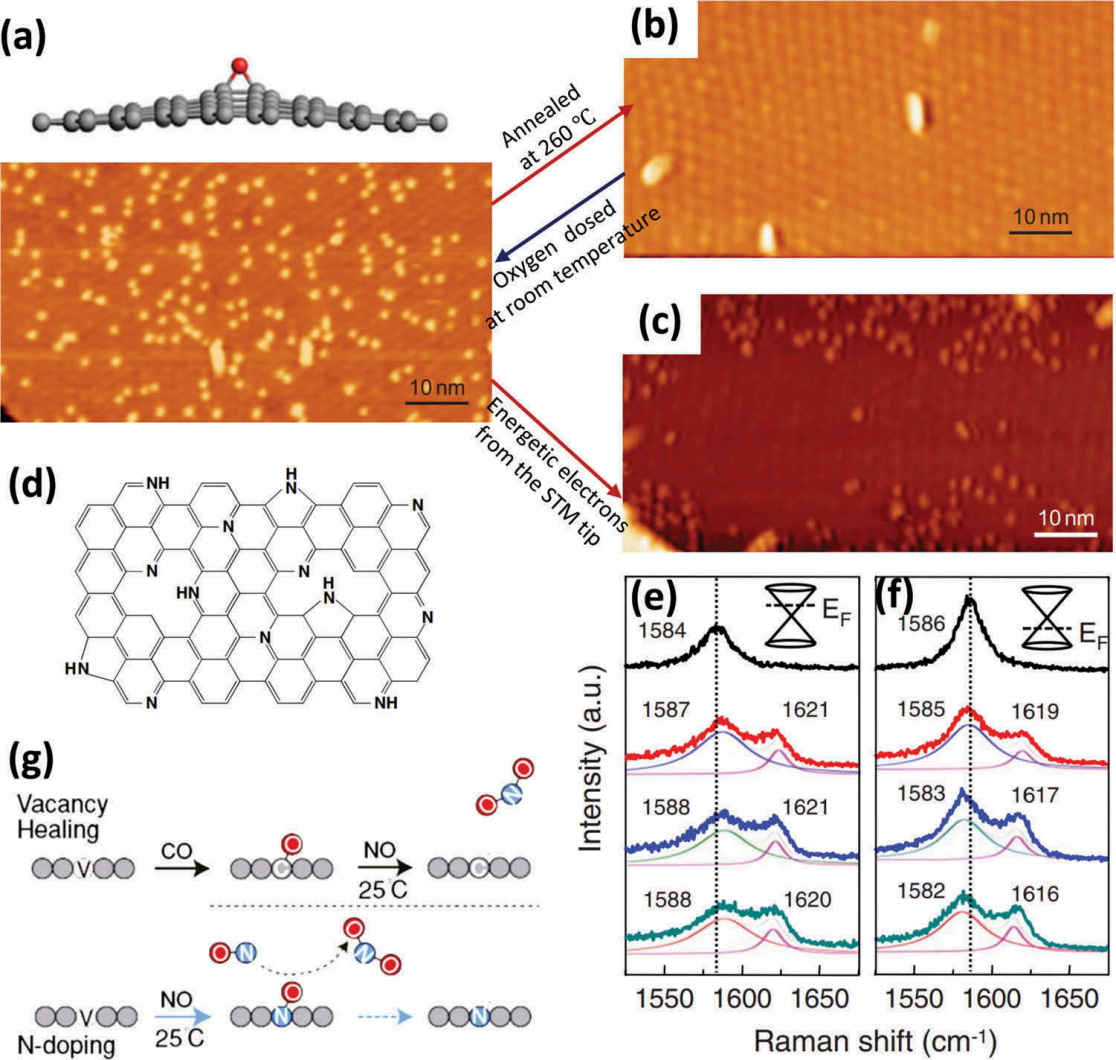
(a) Configuration of chemisorbed oxygen on graphene sheet, which corresponds to bright protrusions in bottom Auger electron spectroscopy (AES) image [345]. STM images of UHV oxidized epitaxial graphene after (b) annealing at 260 °C and (c) reversibly desorbed by injecting electrons from the STM tip at a sample bias of + 4V and tunneling current of 1 nA [345]. (d) Schematic of postulated nitrogenation on graphene [346]. The evolution of G peak upon plasma exposure for graphene with initial Fermi level lying in (e) conduction band and (f) valance band, respectively. The dashed lines indicate the G peak position of pristine graphene [346]. (g) Schematic view of the vacancy healing and N-doping processes of graphene by chemical reactions [331] (reused with permissions from [345] Copyright © 2012, Springer Nature, [346], Rights managed by AIP Publishing, and [331] Copyright ©2011 American Physical Society.).

The reaction of carbon atoms in the graphene sheet with other species can lead to the formation of vacancies or sp^3^ defects. Though the room-temperature reaction is limited by the high-inertness of pristine graphene, strong oxidizing acids (e.g. HNO_3_ and H_2_SO_4_) used in Hummers method can easily react with graphene and attach chemical groups (e.g. oxygen and hydroxyl and carboxyl) to the graphene surface. Due to the chemical inhomogeneity and irreversibility of the resulting GO, an alternative approach using atomic oxygen in ultrahigh vacuum is presented for reversible and uniform oxidation of epitaxial graphene on SiC(0001) [345]. Specifically, the oxidation degree of epitaxial graphene or the density of chemisorbed oxygen (see Figure 19(a)) can be readily tuned by controlling the duration of atomic oxygen exposure. In addition, the chemisorbed oxygen on epitaxial graphene can be reversibly removed by annealing the oxidized surface at 260 °C as well as by energetic electrons from the STM tips, as shown in the STM images in Figure 19(b,c).

Plasma treatment in ammonia gas are commonly used in efforts to dope graphene by nitrogen [287,346]. The nitrogen-containing radicals can readily form covalent bonds with the carbon lattice and retain stable in the post-annealing process. Figure 19(d) shows a typical schematic of postulated nitrogenation on graphene. In plasma treatment of graphene by using a nitrogen/hydrogen gas mixture, an elevated substrate temperature was observed to cause a reduction of defects, and most of the nitrogen atoms were verified to be pyridinelike in carbon networks [347]. Typically, covalent functionalization with amine groups usually occurs at the edges or defect sites of the graphene layer [348]. Besides, substitution of carbon atoms by NH radicals in graphene lattice can cause more efficient N-doping than similar reaction in pentagonal rings (pyrrole-like N). The nitrogen-doping level can be fine-tuned by controlling the exposure time and flow rate, and monitored by Raman spectroscopy (G mode, see Figure 19(e,f)) and transport measurements. Akada et al. [349] found that nitrogen atoms doped at a graphitic site (inside the graphene) lower the work function, while nitrogen atoms at a pyridinic or a pyrrolic site (edge of graphene) increase the work function, and they suggested that the work function of graphene can be tuned from 4.3 eV to 5.4 eV by adjusting plasma treatment time and the amount of initial defects. Further, thermal annealing in ammonia environment allows efficient synthesis of N-doped rGO sheets with fairly high-conductivity [350].

Diels−Alder or other cycloaddition reactions are effective approaches for surface modification of graphene by converting sp^2^ into sp^3^ orbitals. Daukiya et al. [332] successfully performed cycloaddition reactions on graphene by depositing maleimide derivative molecules at room temperature. Experimental results indicate that both (1,4) and (1,2) cycloadditions are possible on freestanding graphene, but only the (1,2) cycloaddition could be activated for SiC (0001)-supported graphene. Besides, the covalently graft of the molecules to graphene resulted in the breakage of the sp^2^ conjugation of carbon atoms and the generation of local sp^3^ bonds. Moreover, the grafted molecules perturb the graphene lattice, and consequently an anisotropic standing-wave pattern is generated due to the (1,2) cycloaddition. In their experiments, the formation of covalent bonds was ascertained by the increased sp^3^ components in the XPS spectrum, while the standing wave patterns were visualized by STM.

On the other hand, disorders in graphene lattice can be reconstructed via chemical reactions between defective graphene sheet and foreign species. Wang and Pantelides [331] carried out studies on the controllable vacancy healing and nitrogen doping of defective graphene by using first-principles MD simulation. By following the chemical reaction schemes in Figure 19(g), the existing vacancies in graphene layer can be healed by exposure to CO. The extra oxygen atoms are removed by the subsequent exposure to NO molecules, which leads the formation of NO_2_. In addition, sequential creation of vacancies (e.g. irradiation of energetic particles) and sequential exposure to NO molecules make it is possible to achieve fine control of N-doping.

### 4.4. Strain treatment

Atomic thickness of graphene makes it amenable to external influences, including mechanical deformation. It is intuitive that strain can cause distortion or other defects to the hexagonal lattice, thus changing the electronic band structure of graphene. Pereira and Castro Neto [351] demonstrated that a stain-induced gauge field can be easily tailored to generate confined surface states, quantum wires and electron beam collimation in graphene. Whereas wrinkles are generated when graphene experiences a uniaxial exterior force, crumples are spontaneously formed as a consequence of multidirectional forces. These strain-induced out-of-plane deformations, if controllable, may be used to tune the electrical and mechanical properties of graphene. The required strains can be created by exploiting difference in thermal expansion of graphene and a substrate [198], by adhering graphene on profiled substrates, by using suspended graphene and by depositing graphene over triangular trenches [352].

**Figure 20.**
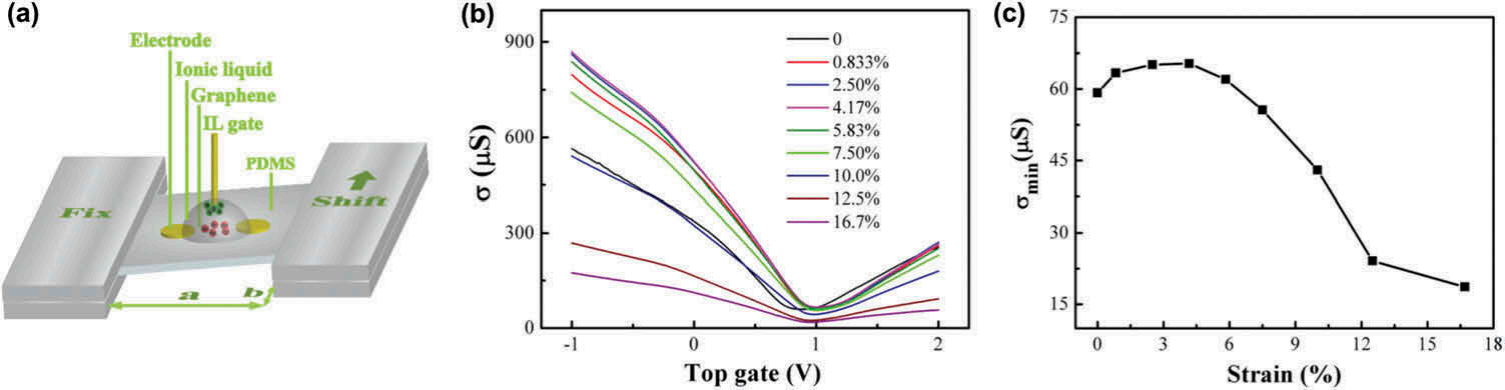
(a) Schematic of the strain device: The red balls represent negative ions, and the green balls represent positive ions of ionic liquid (IL). PDMS stands for polydimethylsiloxane. (b) Transfer characteristics of the ionic liquid gated graphene under different strains. (c) Conductance of the Dirac points under different strains [334] (reused with permission from [334] Rights managed by AIP Publishing.).

Moreover, strain can be applied to a defective lattice for tailoring the properties of graphene. Kumar and Guo [333] used atomistic quantum transport numerical simulations to exam the modification of electrical transport properties for polycrystalline graphene, and to evaluate the impact of strain on the GBs. In their study, the topological structure of GBs determines the modulation of transport gap and electrical conductance. More specifically, the symmetric GBs were insensitive to strain, while the asymmetric-metallic (semiconducting) GBs experienced a metal-to-semiconductor (semiconductor-to-metal) transition. He et al. [334] developed a strain device (see Figure 20(a)) for investigating the influence of shear strain on the transfer characteristics of CVD graphenes. Their experimental results indicate that as the strain increases, the conductance of Dirac point and carrier mobility increases accordingly at low strain (below ~ 3%), but decreases at larger strain, as seen in Figures 20b and 20c. Further, the coactions of the shear strain enhanced transverse conducting channels and the grain boundaries induced strong scattering to carriers are responsible for such behavior.

## 5. Summary

This article describes the structure of graphene from a fundamental perspective, explaining in detail the formation of the honeycombed structure and studying the electronic band structure of graphene via tight-binding approximation. The characterization methods for edge orientations, number and stacking arrangements of graphene layers, and their effects on the properties of graphene are also discussed. To satisfy various demands in practical applications, disorders (i.e. corrugations, topological defects, vacancies, adatoms and sp^3^ defects) are usually deliberately introduced into the structure of graphene. The configuration, formation of these disorders and their influences are systematically introduced. Various approaches (i.e. particle irradiation, thermal annealing, chemical reaction and strain treatment) for defects modulation in graphene are discussed at the end of this article. This review is expected to facilitate the understanding of the structure of graphene and its contained disorders, and consequently assist in investigations into graphene modification.
